# Supplementary figures and images for: DSCAM gene triplication causes excessive GABAergic synapses in the neocortex in Down syndrome mouse models
Source: PLoS Biol. 2023 Apr 20;21(4):e3002078. doi: 10.1371/journal.pbio.3002078 (PMC10118173; doi:10.1371/journal.pbio.3002078)

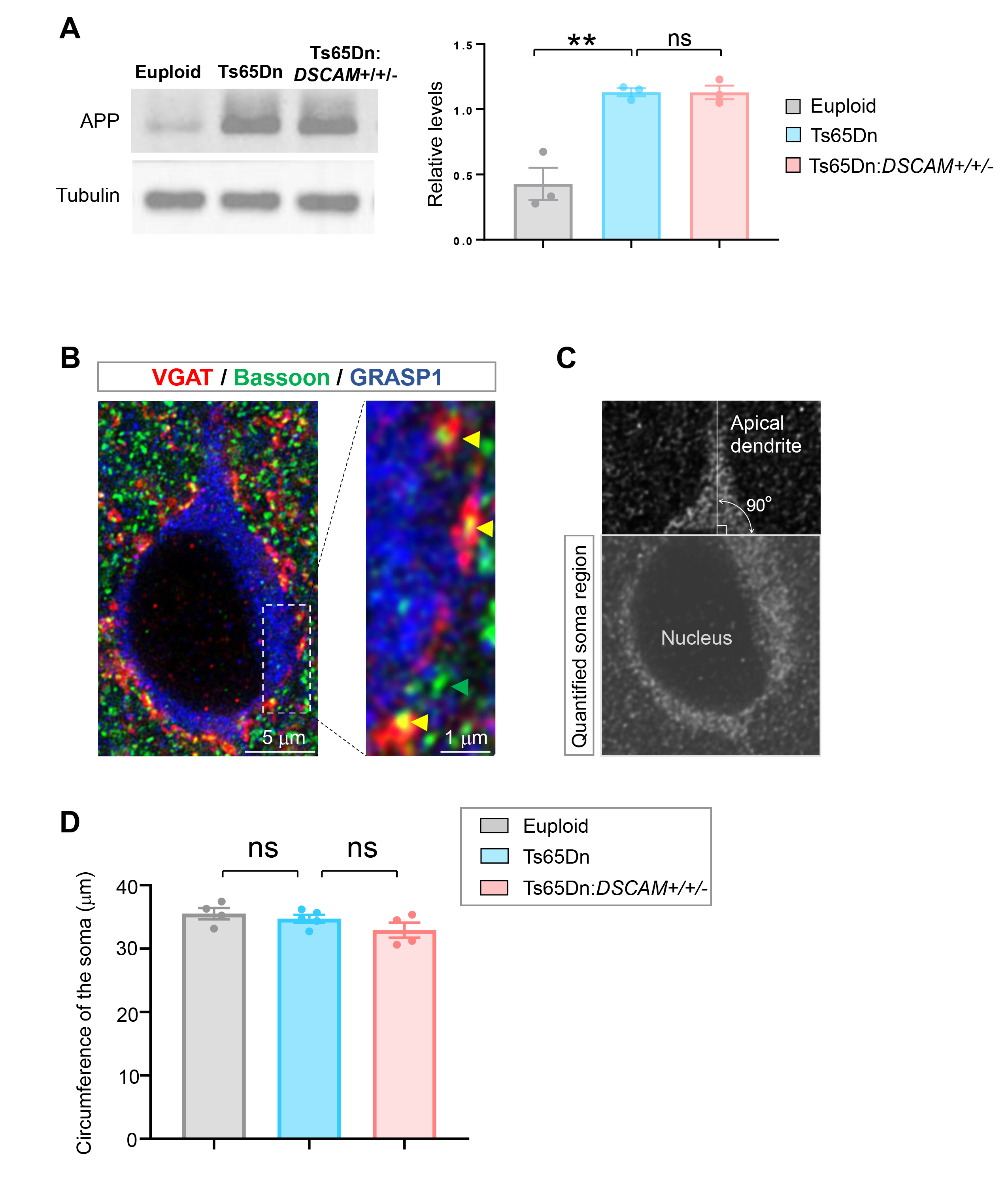

Supplement: S1 Fig — (A) Normalizing DSCAM gene dosage does not change the increased level of APP in Ts65Dn cortices. Shown are representative western blots (left) and quantifications (right) of neocortical samples from each indicated genotype. Each dot in the bar chart represents the sample from 1 mouse. (B) Representative confocal image of a PyN in layer II/III in the ACC. The soma is labeled by anti-GRASP1 (blue). The presynaptic active zones are labeled by anti-Bassoon (green). The GABA vesicles in presynaptic terminals of GABAergic neurons are labeled by anti-VGAT (red). The perisomatic Bassoon+ puncta that overlap with VGAT+ puncta were quantified as GABAergic boutons (yellow arrowheads). The green arrowhead indicates the VGAT-independent perisomatic Bassoon+ puncta. (C) To define the soma region for quantifying perisomatic GABAergic boutons, we drew a line that is both perpendicular to the apical dendrite and tangent to the edge of the PyN nucleus and then quantified GABAergic boutons in the GRASP1+ area below the line. (D) The soma size of PyNs in the ACC is not affected in Ts65Dn or Ts65Dn:DSCAM+/+/− mice. The quantifications show the mean circumference of the soma. Each data point is the mean in a mouse. Statistical tests are one-way ANOVA for multigroup comparisons and post hoc Student t tests for pair-wise comparisons. **: p < 0.01; ns: not significant (p > 0.05). Unless specified, mean ± SEM is shown in the figures. The data underlying this Figure can be found in https://doi.org/10.5281/zenodo.7714234. ACC, anterior cingulate cortex; APP, amyloid precursor protein; DSCAM, Down syndrome cell adhesion molecule; PyN, pyramidal neuron. (TIF) [file pbio.3002078.s001.tif]

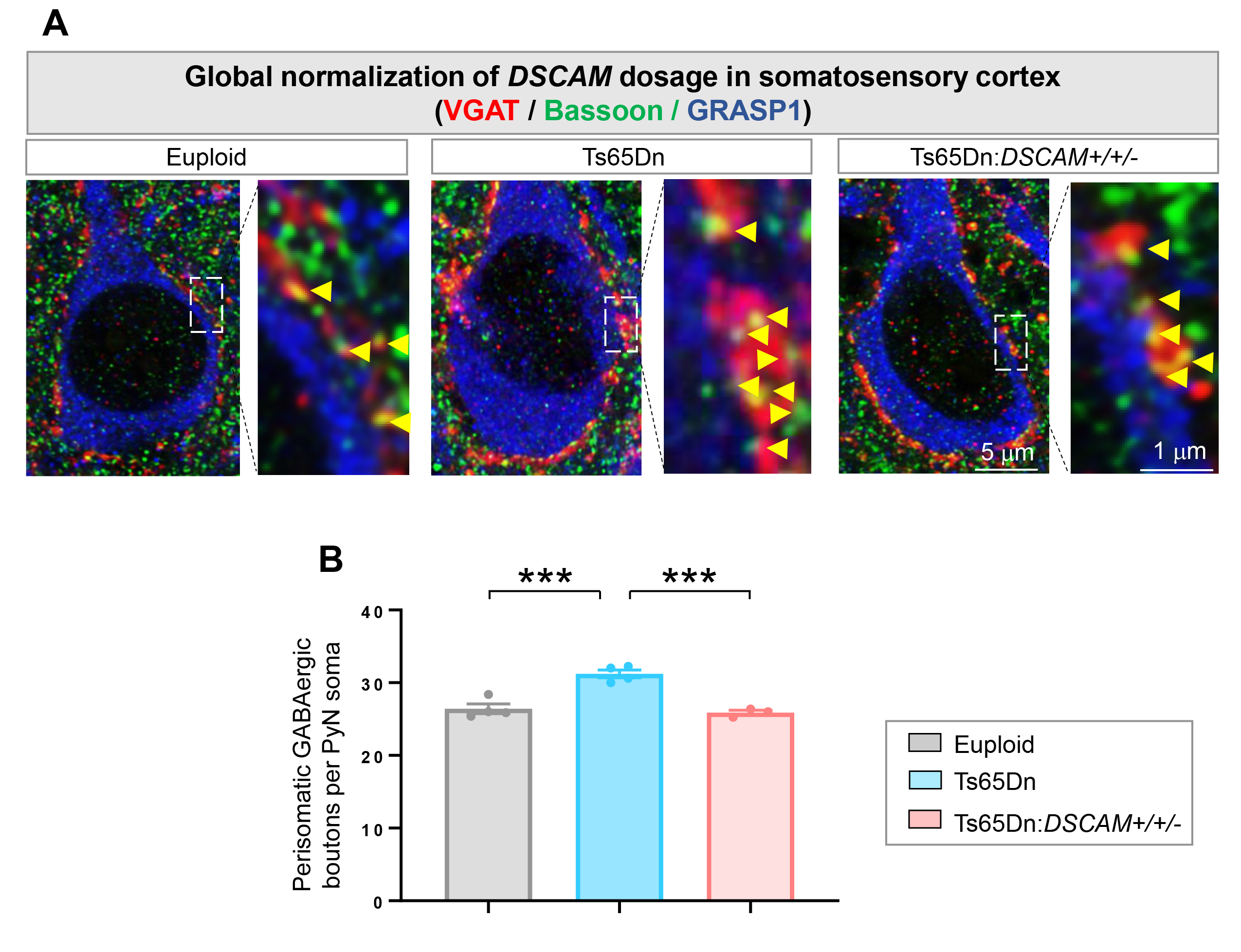

Supplement: S2 Fig — (A) Representative images of perisomatic GABAergic boutons innervating PyNs in layer II/III of the somatosensory cortex of euploid (wild-type), Ts65Dn, and Ts65Dn:DSCAM+/+/−. The right panel in each genotype group is the magnified view of the regions boxed by dotted lines in the left panel. The soma and proximal dendrites of PyNs were labeled by GRASP1. Yellow arrowheads point to GABAergic boutons as indicated by Bassoon+ puncta that overlap with VGAT+ puncta. (B) Quantification of the number of perisomatic GABAergic boutons per PyN in the somatosensory cortex. Each data point in the chart represent the mean in 1 mouse. One-way ANOVA for multigroup comparisons and post hoc Student t tests for pair-wise comparisons. ***: p < 0.001. The data underlying this Figure can be found in https://doi.org/10.5281/zenodo.7714234. DSCAM, Down syndrome cell adhesion molecule; PyN, pyramidal neuron. (TIF) [file pbio.3002078.s002.tif]

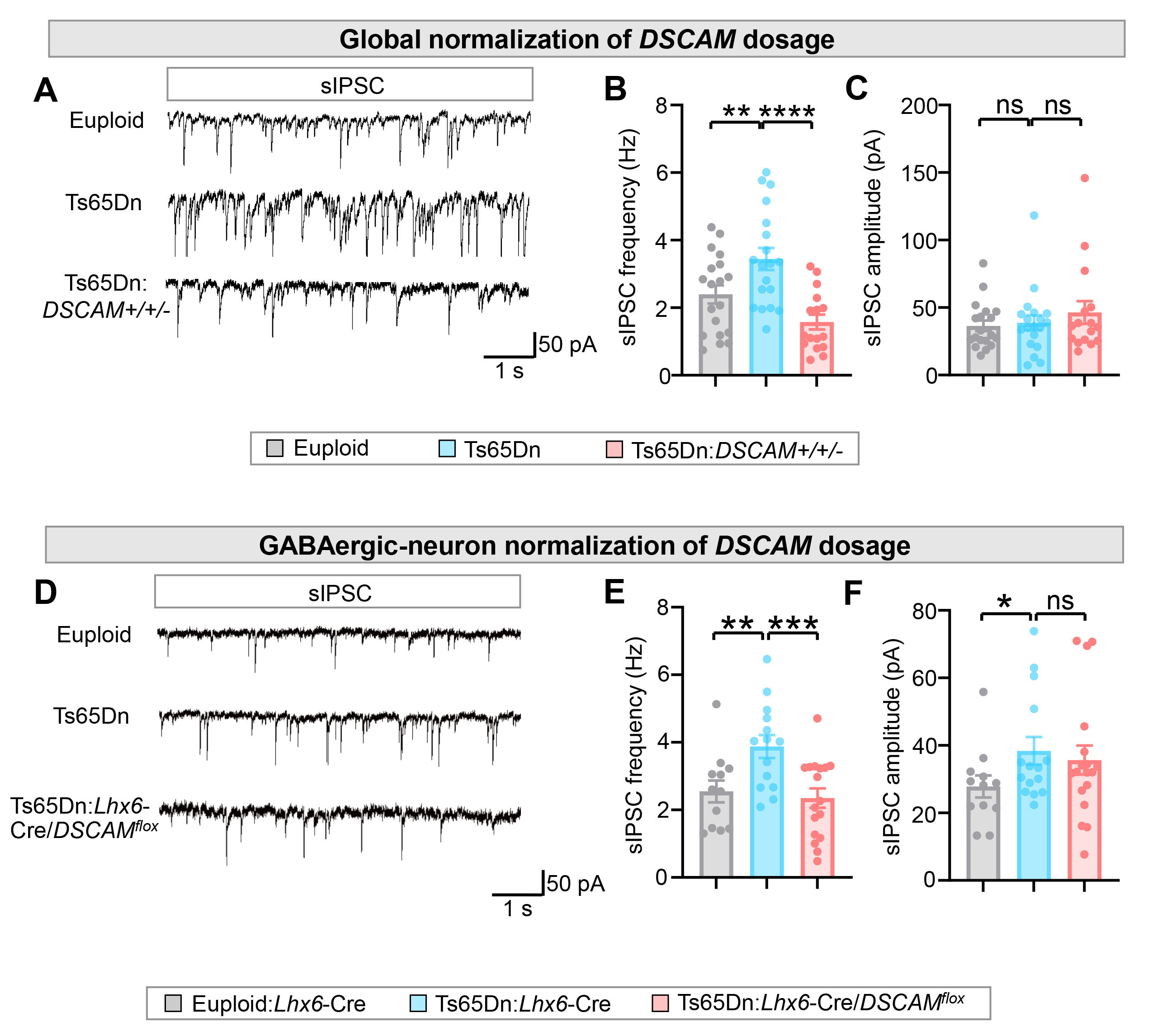

Supplement: S3 Fig — (A) Representative traces of sIPSCs from PyNs in layer II/III of the ACC in the euploid, Ts65Dn and Ts65Dn:DSCAM+/+/− brain slices. (B, C) Quantification of sIPSC frequency (B) and amplitude (C). Approximately 2–4 PyNs were recorded for each mouse. A total of 6 euploid, 7 Ts65Dn, and 6 Ts65Dn:DSCAM+/+/− mice were analyzed. N: 19 for euploid, 19 for Ts65Dn, and 16 Ts65Dn:DSCAM+/+/−. (D) Representative traces of sIPSCs from PyNs in layer II/III of the ACC in euploid:Lhx6-Cre, Ts65Dn:Lhx6-Cre, and Ts65Dn:Lhx6-Cre:DSCAMflox brain slices. DSCAM gene dosage was normalized in GABAergic neurons in the Ts65Dn:Lhx6-Cre:DSCAMflox mice. (E, F) Quantification of sIPSC frequency (E) and amplitude (F). For each mouse, 2–4 PyNs were recorded. A total of 4 euploid control, 4 Ts65Dn, and 4 Ts65Dn:Lhx6-Cre:DSCAMflox mice were analyzed. N: 12 for euploid, 14 for Ts65Dn, and 17 Ts65Dn:DSCAM+/+/−. One-way ANOVA for multigroup comparisons and post hoc Student t tests for pair-wise comparisons. *: p < 0.05; **: p < 0.01; ***: p < 0.001; ****: p < 0.0001; ns: not significant (p > 0.05). The data underlying this Figure can be found in https://doi.org/10.5281/zenodo.7714234. ACC, anterior cingulate cortex; DSCAM, Down syndrome cell adhesion molecule; PyN, pyramidal neuron; sIPSC, spontaneous inhibitory postsynaptic current. (TIF) [file pbio.3002078.s003.tif]

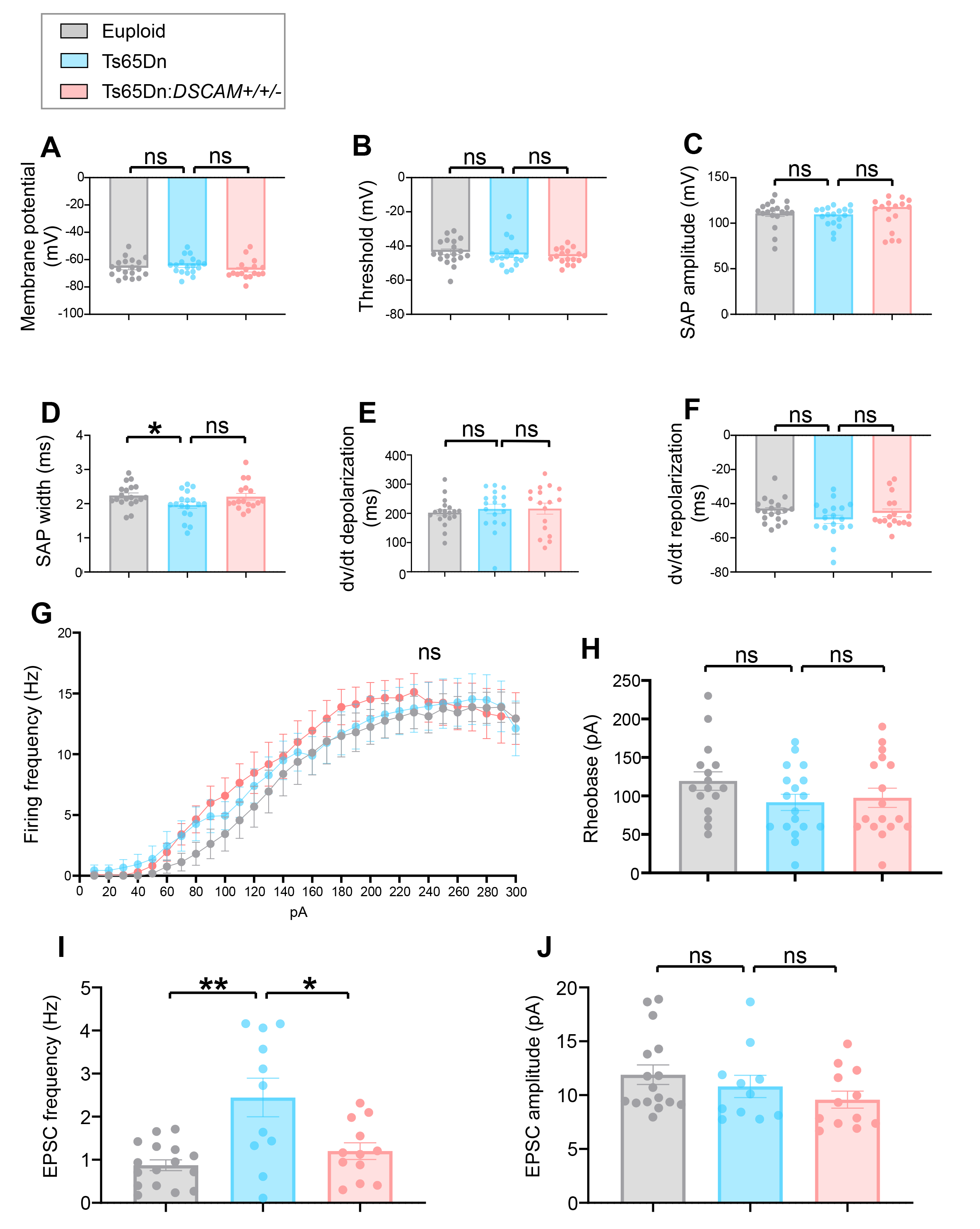

Supplement: S4 Fig — Quantification of electrophysiology parameters of PyNs in the ACC in euploid (gray), Ts65Dn (light blue), and Ts65Dn:DSCAM+/+/− (pink) brain slices. Data shown as mean ± SEM. Kruskal–Wallis test with post hoc Mann–Whitney tests for two-group comparisons, except for (G). *: p < 0.05; **: p < 0.01; ns: not significant (p > 0.05). (A-F) Quantifications of membrane potential (mV) (A), threshold (mV) (B), SAP amplitude (mV) (C), SAP half-width (ms) (D), the depolarization velocity of SAP (dv/dt) (E), and repolarization velocity of SAP (dv/dt) (F). Cell numbers: 19 for euploid, 18 for Ts65Dn, and 17 Ts65Dn:DSCAM+/+/−. (G) Curves showing the relationship between the average firing frequencies of evoked AP (Hz) and the currents (pA) in PyNs. Two-way ANOVA, Tukey’s multiple comparisons test. ns: p > 0.05. (H) Rheobase (pA) of PyNs in (G). One-way ANOVA with post hoc Kruskal–Wallis test. ns: p > 0.05. N: 16 for euploid, 18 for Ts65Dn, and 17 Ts65Dn:DSCAM+/+/−. (I, J) Quantification of sEPSC frequency (I) and amplitude (J). For each mouse, 2–4 PyNs were recorded. The data underlying this Figure can be found in https://doi.org/10.5281/zenodo.7714234. ACC, anterior cingulate cortex; PyN, pyramidal neuron; SAP, single action potential; sEPSC, spontaneous excitatory postsynaptic current. (TIF) [file pbio.3002078.s004.tif]

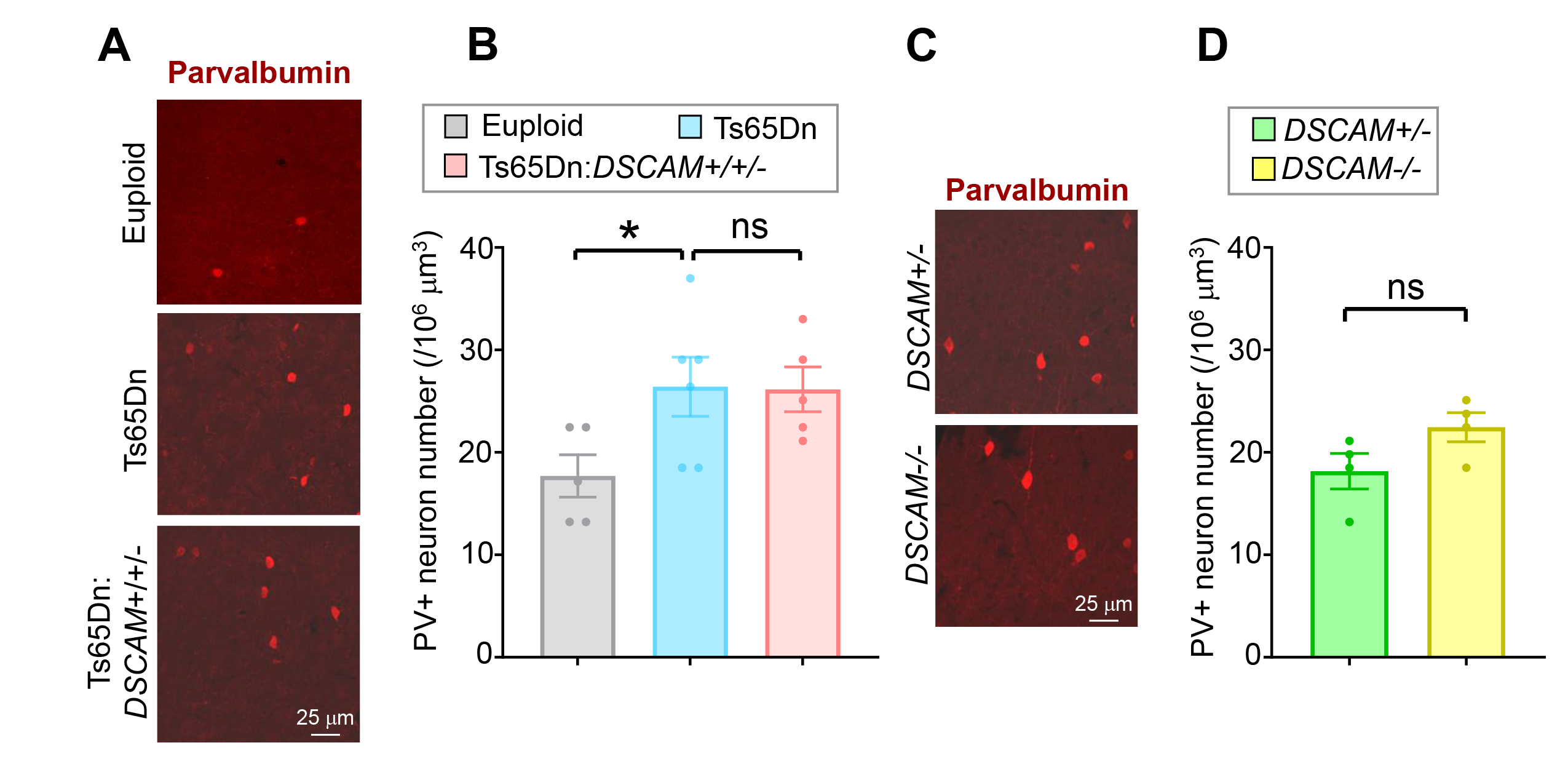

Supplement: S5 Fig — (A, B) DSCAM overexpression in Ts65Dn mice does not affect the number of GABAergic neurons in the neocortex. Brain sections from P28 mice were immunostained with anti-PV. Representative images are shown in (A), and quantifications of the density of PV+ neurons are shown in (B). Each dot represents the value from 1 imaging field that is 252.4 μm (width) × 200 μm (length) × 5 μm (thickness). Images were collected from layer II/III of the ACC. Three fields in each mouse were randomly selected for imaging. A total of 5 euploid, 6 Ts65Dn, and 5 Ts65Dn:DSCAM+/+/− mice were analyzed. One-way ANOVA for multigroup comparisons and post hoc Student t tests for pair-wise comparisons. *: p < 0.05; ns: not significant (p > 0.05). (C, D) Loss of DSCAM does not affect the number of GABAergic neurons in the ACC. Representative images and quantifications are shown in (C) and (D), respectively. A total of 4 DSCAM+/− and 4 DSCAM−/− mice were analyzed. Student t test. ns: not significant (p > 0.05). The data underlying this Figure can be found in https://doi.org/10.5281/zenodo.7714234. ACC, anterior cingulate cortex; DSCAM, Down syndrome cell adhesion molecule; PV, parvalbumin; P28, postnatal day 28. (TIF) [file pbio.3002078.s005.tif]

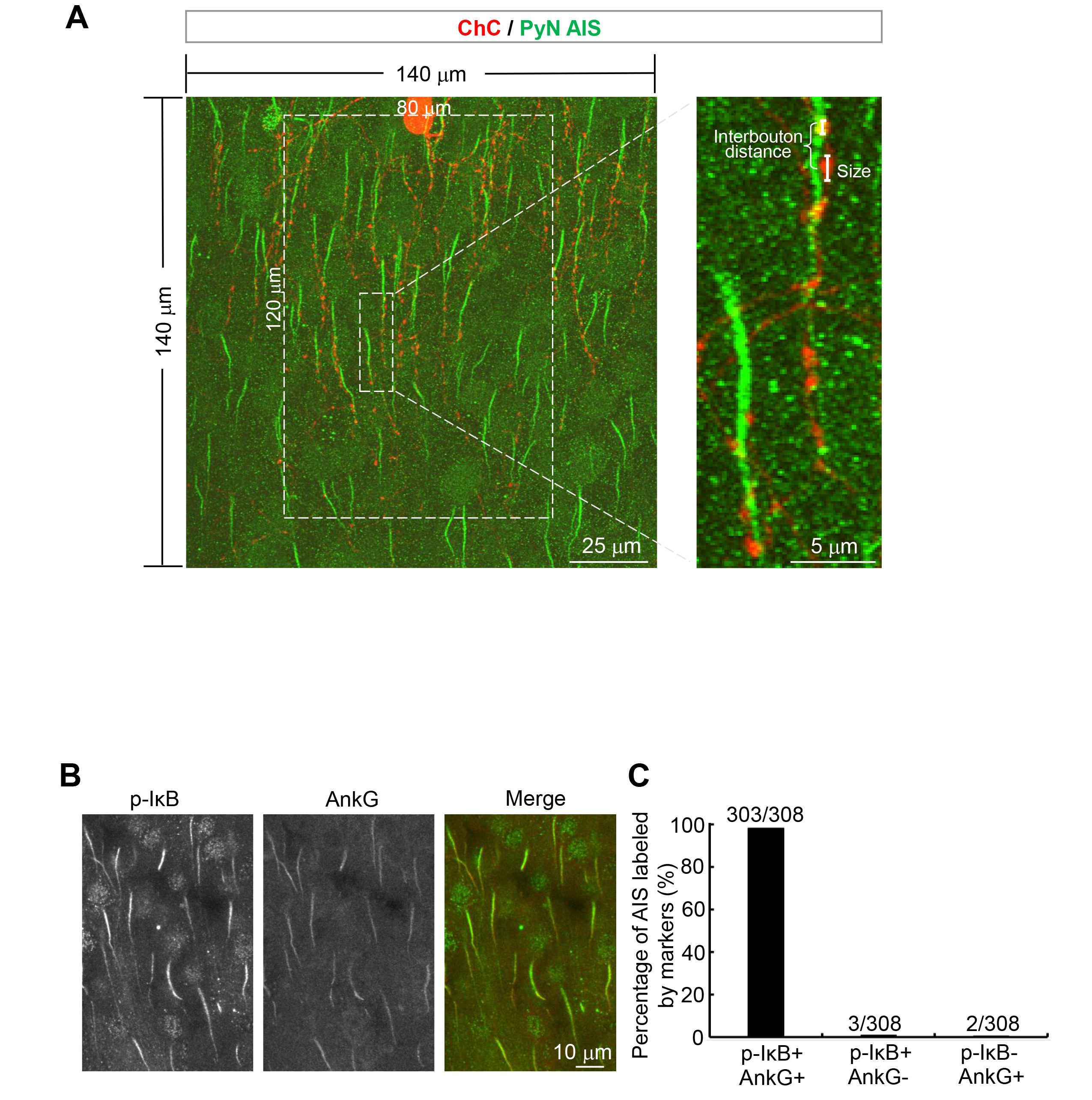

Supplement: S6 Fig — (A) ChCs were sparsely labeled by tdTomato (red), and AISs of PyNs were labeled by immunostaining with anti-phospho-IκB (green). Confocal image stacks (0.3 μm z-steps for 100 steps) were maximally projected along the z-axis. A region of 120 μm (length) × 80 μm (width) with the cell body in the top middle was quantified. Cartridges and boutons that colocalized with AIS were quantified. Cartridge number was defined as the number of cartridges within this region. Cartridge length was defined as the distance from the first to the last bouton that colocalizes with the AIS in that cartridge. Bouton size is defined as the length of bouton in parallel to AIS. Interbouton distance is defined as the distance between 2 neighboring boutons. (B, C) Phospho-IκB and AnkG shows equal fidelity in labeling neocortical AIS. (B) AIS in layer II/III ACC was colabeled by phospho-IκB (green) and AnkG (red). Shown are maximal projection of confocal image stacks (1 μm z-steps X 7 steps). (C) Quantification of the percentage of AIS that is labeled by phospho-IκB and/or AnkG. A total of 308 AIS were quantified, among which 303 were colabeled by phospho-IκB and AnkG. The data underlying this Figure can be found in https://doi.org/10.5281/zenodo.7714234. ACC, anterior cingulate cortex; AIS, axon initial segment; ChC, chandelier cell; PyN, pyramidal neuron. (TIF) [file pbio.3002078.s006.tif]

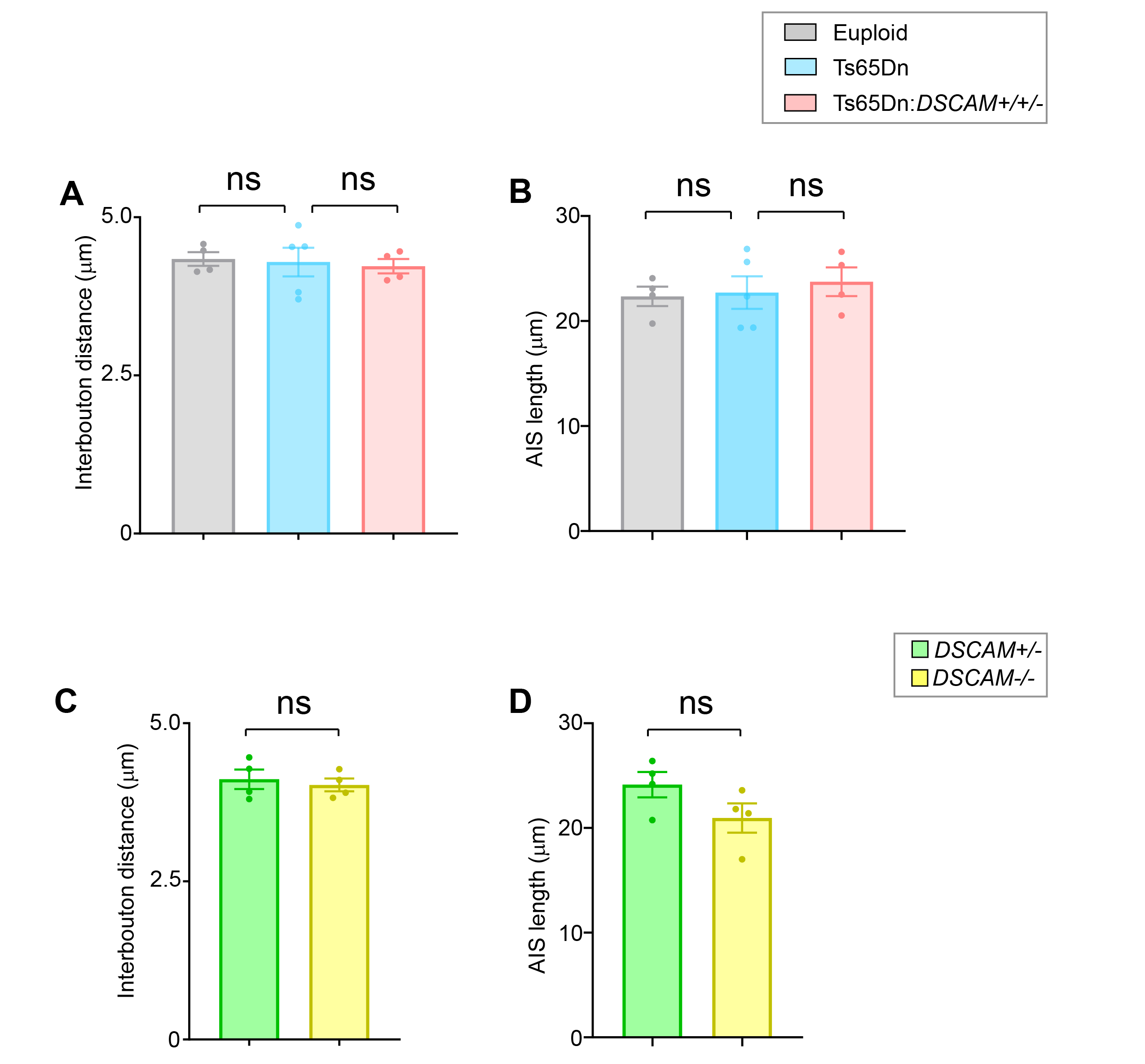

Supplement: S7 Fig — (A, B) Neither the average interbouton distance between neighboring boutons nor the AIS length of PyNs was affected in the layer II/III of the ACC of euploid, Ts65Dn, and Ts65Dn:DSCAM+/+/−. One-way ANOVA for multigroup comparisons and post hoc Student t tests for pair-wise comparisons. ns: not significant (p > 0.05). Each data point in the chart represent the mean of 8–10 PyNs in 1 mouse. (C, D) The average interbouton distance between neighboring boutons and the AIS length of PyNs were not significantly different between DSCAM2j/+ (+/−) and DSCAM2j/2j (−/−) mice. Student t tests. Each data point in the chart represent the mean of 4–5 PyNs in 1 mouse. The data underlying this Figure can be found in https://doi.org/10.5281/zenodo.7714234. ACC, anterior cingulate cortex; AIS, axon initial segment; DSCAM, Down syndrome cell adhesion molecule; PyN, pyramidal neuron. (TIF) [file pbio.3002078.s007.tif]

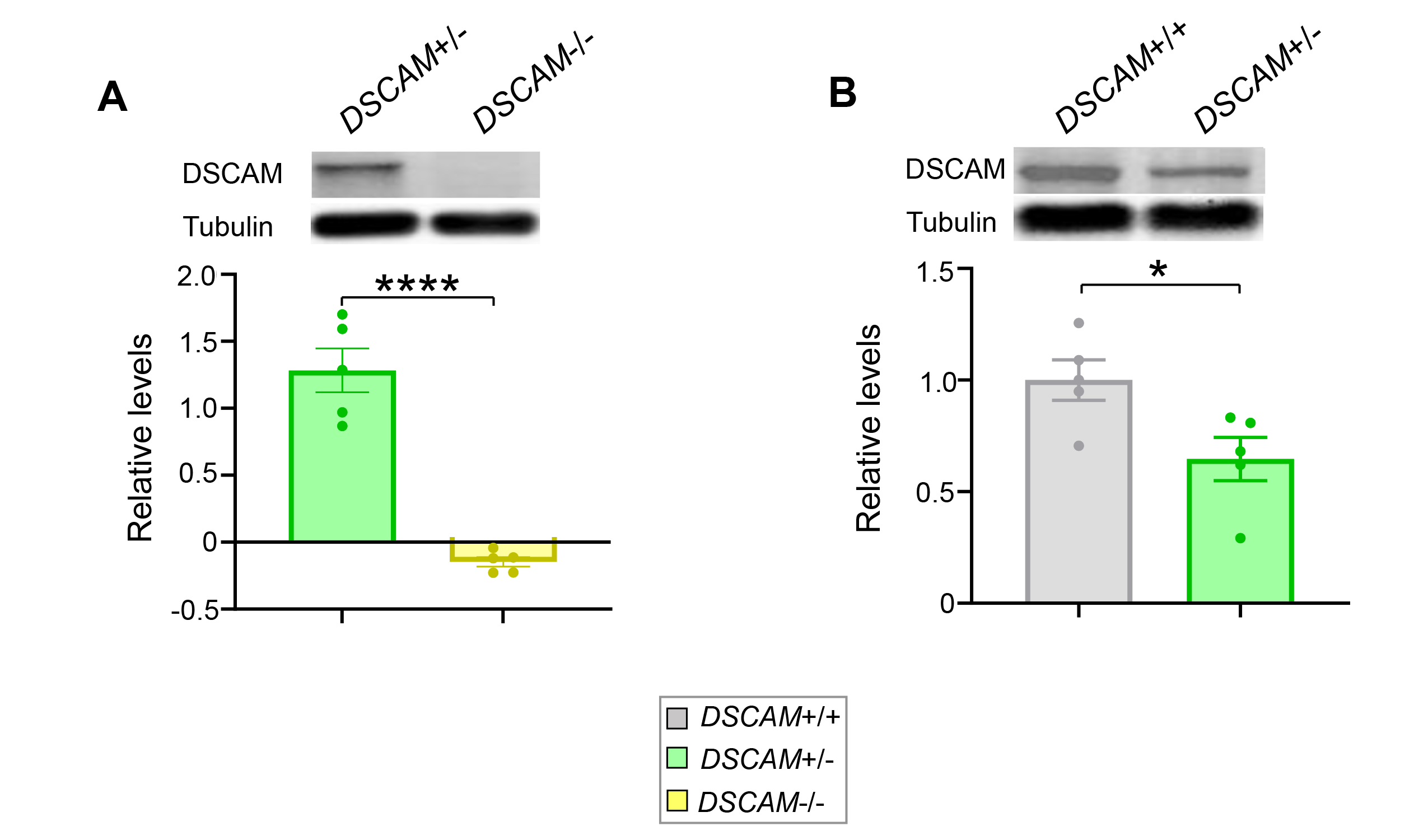

Supplement: S8 Fig — Representative western blots and quantifications of protein samples collected from the somatosensory cortex of DSCAM+/+ (+/+), DSCAM2j/+ (+/−), and DSCAM2j/2j (−/−) mice. DSCAM protein was not detected in DSCAM−/− by western blotting (A). The level of DSCAM protein in DSCAM+/− was about 81% of that in DSCAM+/+ (B). Student t test. *: p < 0.05; ****: p < 0.0001. The data underlying this Figure can be found in https://doi.org/10.5281/zenodo.7714234. DSCAM, Down syndrome cell adhesion molecule. (TIF) [file pbio.3002078.s008.tif]

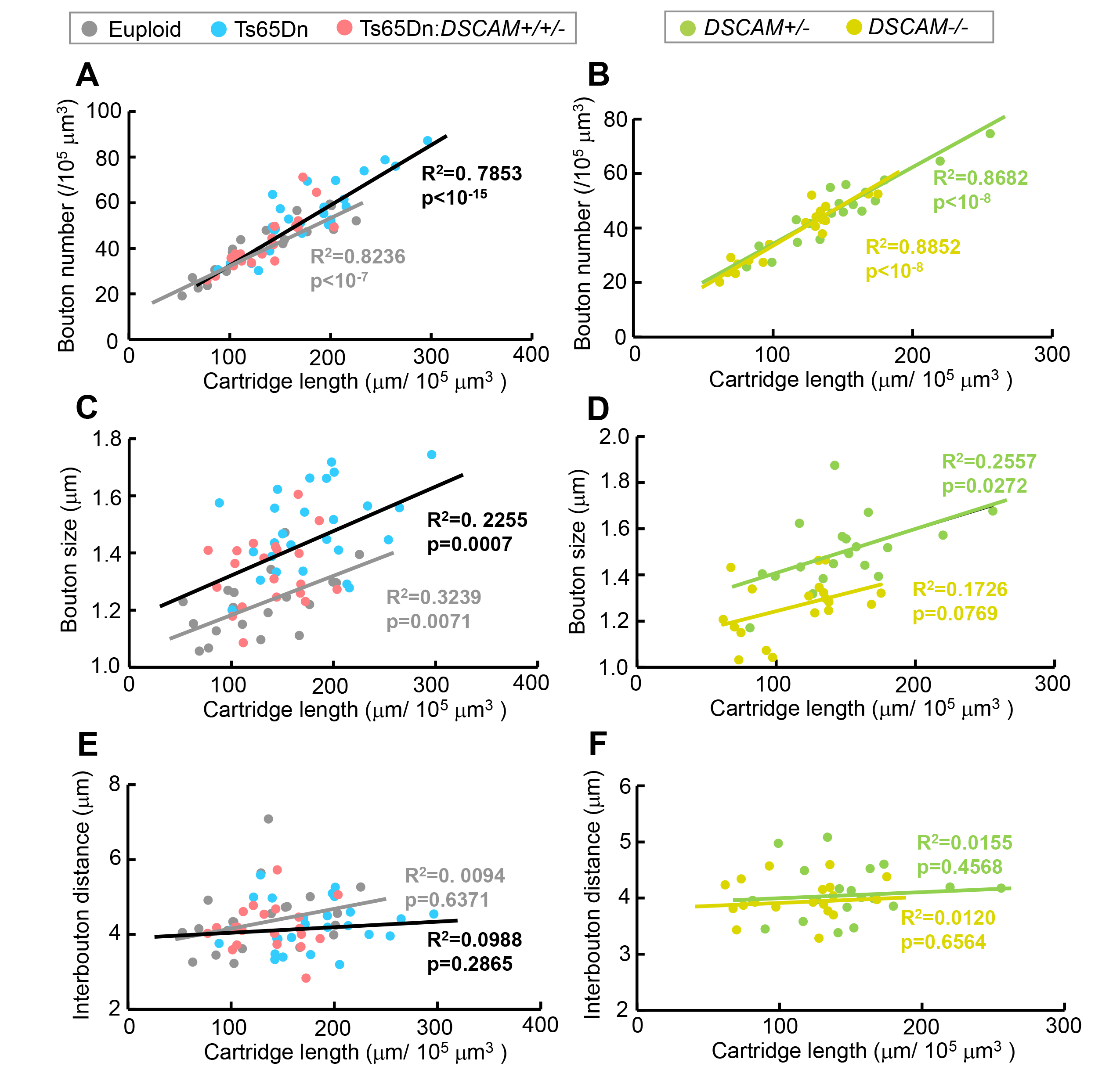

Supplement: S9 Fig — (A) ChC cartridge length and bouton number are strongly correlated in both euploid and Ts65Dn genetic backgrounds. Each dot presents 1 ChC. For each mouse, 4–6 ChCs were analyzed. A total of 4 euploid, 5 Ts65Dn, 4 Ts65Dn:DSCAM+/+/− mice were analyzed. n = 21 for euploid (gray dots), 26 for Ts65Dn (cyan dots), 21 Ts65Dn:DSCAM+/+/− (red dots). R2 and p are calculated for linear regression. The gray line indicates the trend line for gray dots, while the black line is that for blue and coral dots. (B) ChC cartridge length and bouton number are strongly correlated in DSCAM+/− and DSCAM−/− mice. Each dot presents 1 ChC. For each mouse, 4–6 ChCs were analyzed. A total of 4 DSCAM+/− and 4 DSCAM−/− mice were analyzed. N: 19 for DSCAM+/− (green dots) and 19 for DSCAM−/− (yellow dots). (C) ChC cartridge length and bouton size show weak, yet significant, correlation in euploid and Ts65Dn background. Each dot presents 1 ChC. (D) The correlation between cartridge length and bouton number is impaired in DSCAM−/− mice. Each dot presents 1 ChC. R2 is small in both DSCAM+/− and DSCAM−/− mice, suggesting that linear regression only explains a small fraction of the samples. The correction is insignificant in DSCAM−/− mice (p > 0.05). (E, F) ChC cartridge length and interbouton distance shows no significant correlation between any 2 genotypes tested. Each dot presents 1 ChC. The data underlying this Figure can be found in https://doi.org/10.5281/zenodo.7714234. ChC, chandelier cell; DSCAM, Down syndrome cell adhesion molecule. (TIF) [file pbio.3002078.s009.tif]

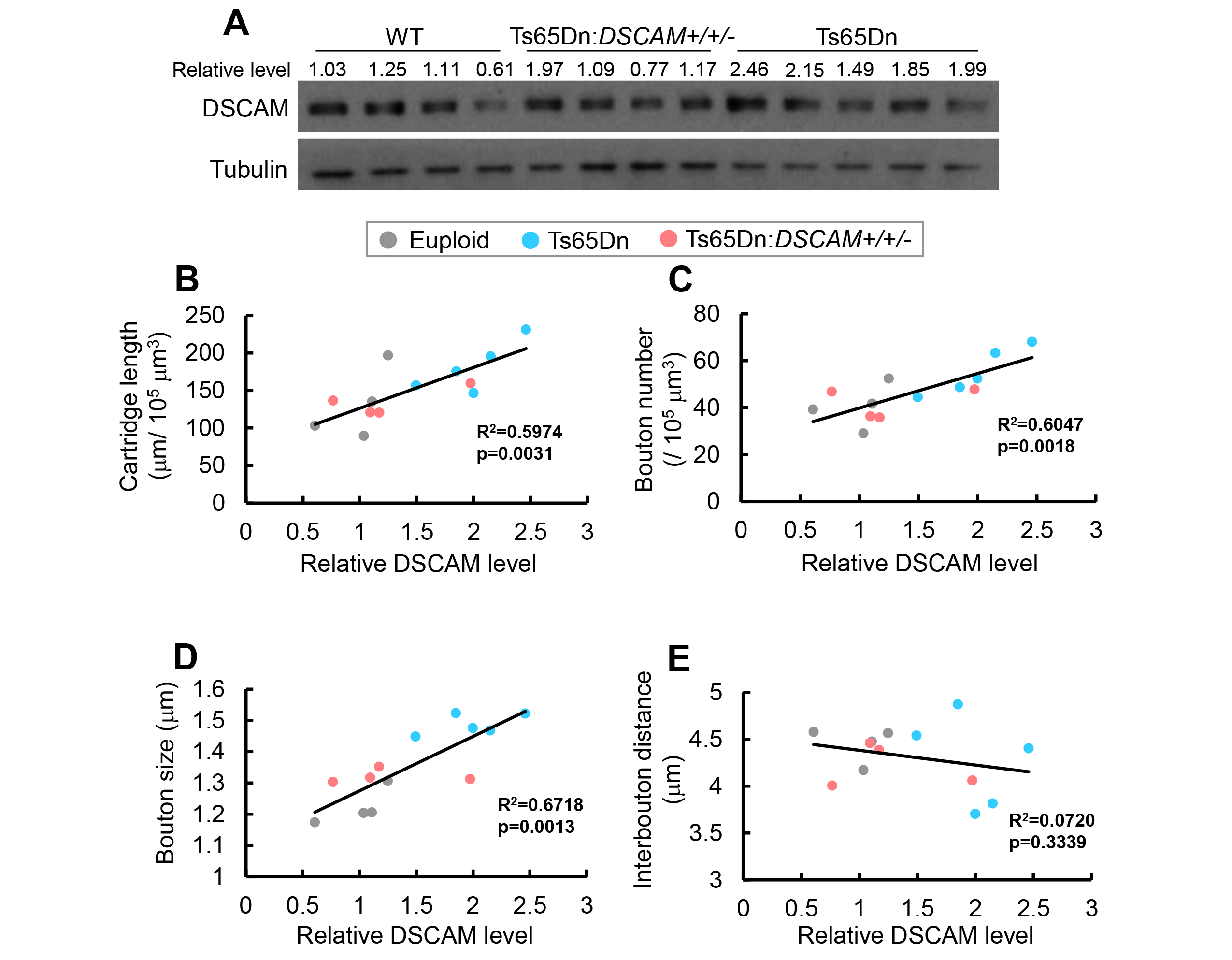

Supplement: S10 Fig — (A) Western blots showing DSCAM levels in the neocortex. The mouse neocortex was taken immediately after perfusion. (B-E) The correlation analyses between DSCAM expression level and ChC cartridge length (B), bouton number (C), bouton size (D), or interbouton distance (E). In (B), the DSCAM level of a mouse is plotted against the mean of the total cartridge length in the volume specified in S1 Fig in this mouse. The average cartridge length is calculated as the average value of 4–6 ChCs sampled in each mouse. A total of 4 euploid (gray dots), 5 Ts65Dn (cyan dots), and 4 Ts65Dn:DSCAM+/+/− (red dots) mice were analyzed. R2 and p are calculated for linear regression. The data underlying this Figure can be found in https://doi.org/10.5281/zenodo.7714234. ChC, chandelier cell; DSCAM, Down syndrome cell adhesion molecule. (TIF) [file pbio.3002078.s010.tif]

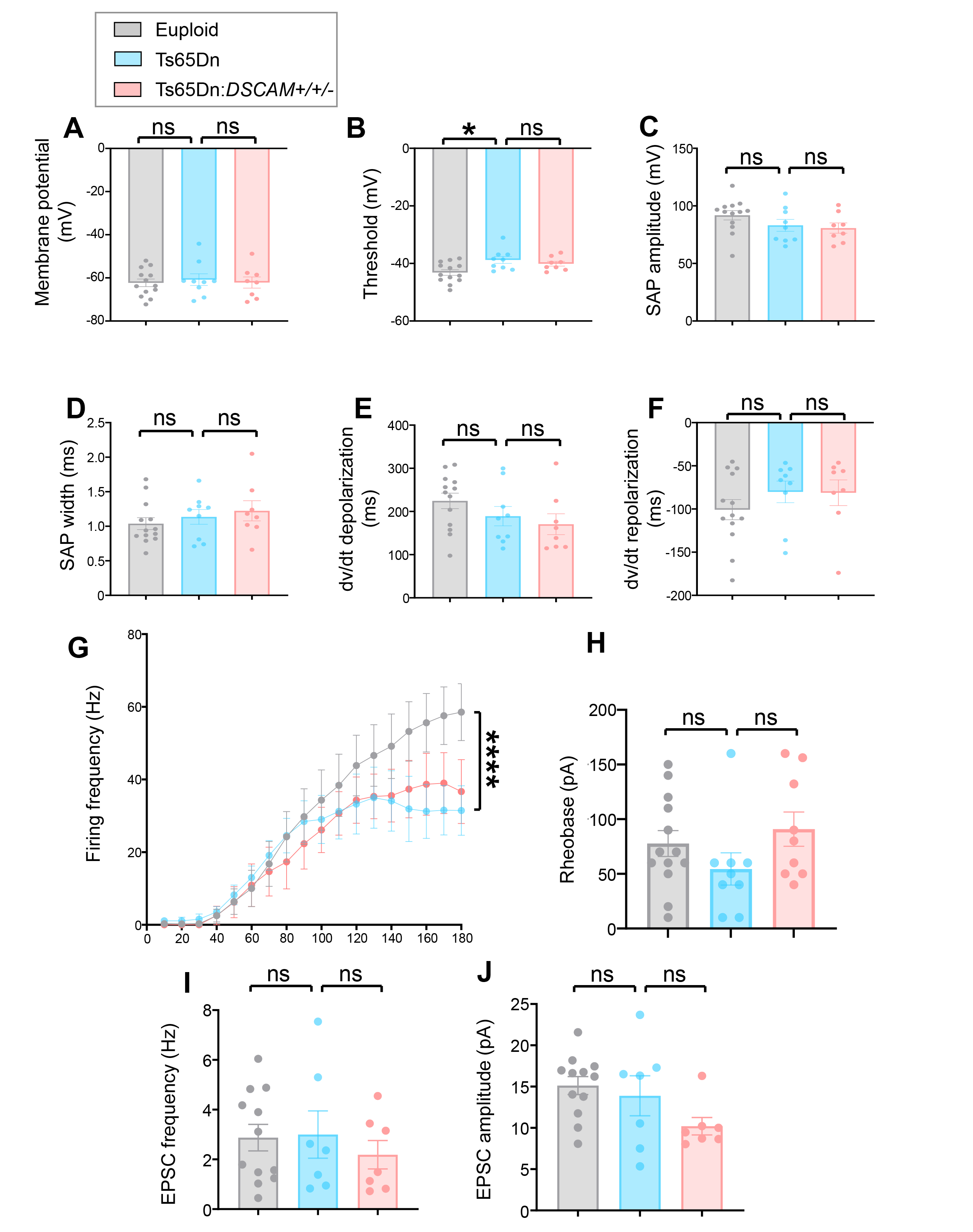

Supplement: S11 Fig — Quantification of electrophysiology parameters of ChCs in the ACC in euploid (gray), Ts65Dn (light blue), and Ts65Dn:DSCAM+/+/− (pink) brain slices. Kruskal–Wallis test with post hoc Mann–Whitney tests for two-group comparisons, except for (G). *: p < 0.05; ns: not significant (p > 0.05). (A-F) Quantifications of membrane potential (mV) (A), threshold (mV) (B), SAP amplitude (mV) (C), SAP half-width (ms) (D), the depolarization velocity of SAP (dv/dt) (E), and repolarization velocity of SAP (dv/dt) (F). Cell numbers: 13 for euploid, 9 for Ts65Dn, and 8 Ts65Dn:DSCAM+/+/−. (G) Curves showing the relationship between the average firing frequencies of evoked AP (Hz) and the currents (pA) in ChCs. Two-way ANOVA, Tukey’s multiple comparisons test. Euploid vs. Ts65Dn: p < 0.0001 (****); Ts65Dn vs. Ts65Dn:DSCAM+/+/−: p > 0.05. (H) Rheobase (pA) from ChCs in (G). Cell numbers: 13 for euploid, 9 for Ts65Dn, and 9 Ts65Dn:DSCAM+/+/−. (I, J) Quantification of sEPSC frequency (I) and amplitude (J). For each mouse, 2–4 PyNs were recorded. Cell numbers: 12 for euploid, 7 for Ts65Dn, and 7 Ts65Dn:DSCAM+/+/−. The data underlying this Figure can be found in https://doi.org/10.5281/zenodo.7714234. ACC, anterior cingulate cortex; ChC, chandelier cell; DSCAM, Down syndrome cell adhesion molecule; SAP, single action potential; sEPSC, spontaneous excitatory postsynaptic current. (TIF) [file pbio.3002078.s011.tif]
