# Supplementary material for: DSCAM gene triplication causes excessive GABAergic synapses in the neocortex in Down syndrome mouse models
Source: PLoS Biol. 2023 Apr 20;21(4):e3002078. doi: 10.1371/journal.pbio.3002078 (PMC10118173; doi:10.1371/journal.pbio.3002078)
Supplement: S1 Raw Images — (PDF) [file pbio.3002078.s012.pdf]

For Fig 1A

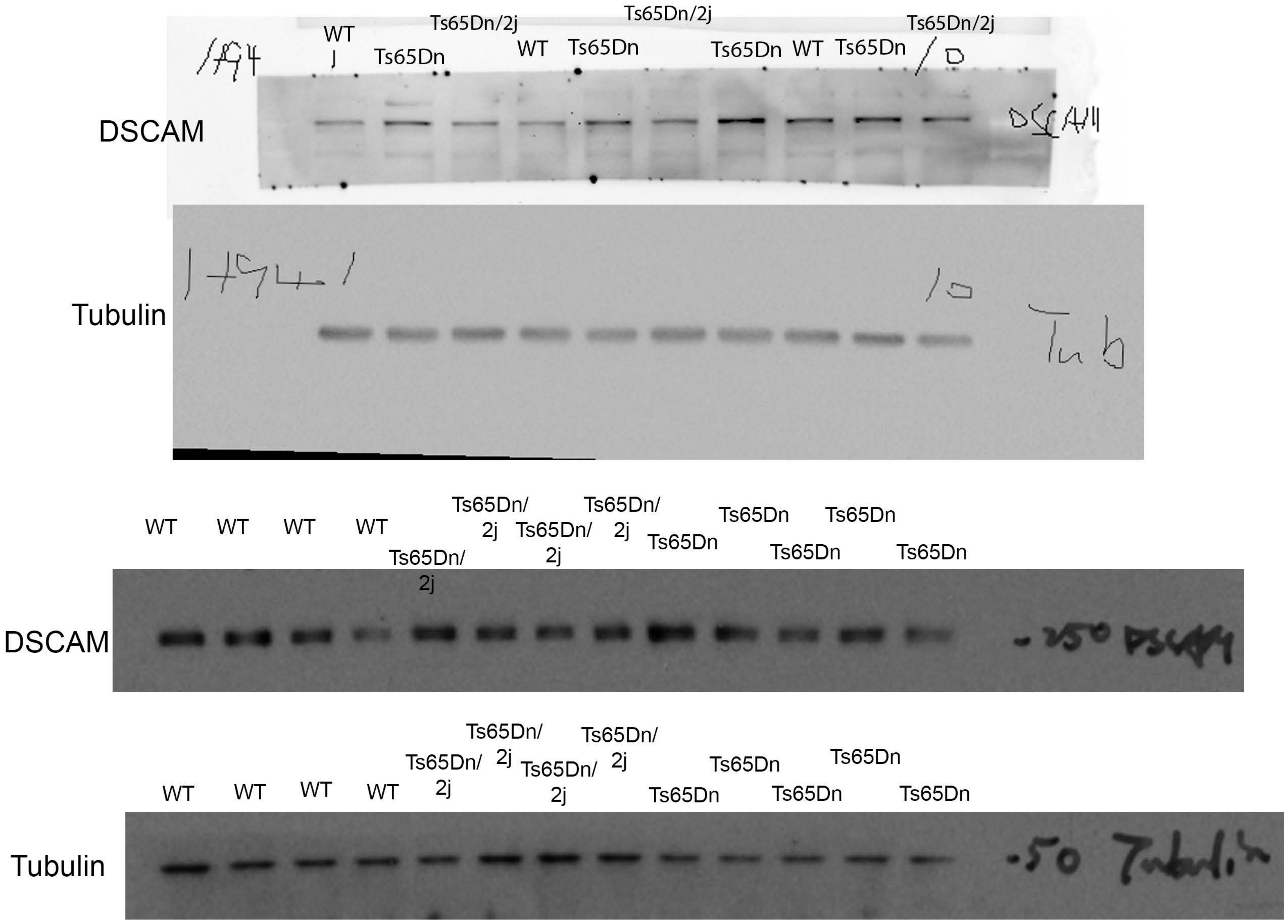
WT
Ts65Dn
Ts65Dn/2j
WT
Ts65Dn
Ts65Dn/2j
Ts65Dn
WT
Ts65Dn
Ts65Dn/2j
DSCAM
Tubulin
Tub
WT
WT
WT
WT
Ts65Dn/2j
Ts65Dn/2j
Ts65Dn/2j
Ts65Dn/2j
Ts65Dn
Ts65Dn
Ts65Dn
Ts65Dn
Ts65Dn
DSCAM
-250
WT
WT
WT
WT
Ts65Dn/2j
Ts65Dn/2j
Ts65Dn/2j
Ts65Dn/2j
Ts65Dn
Ts65Dn
Ts65Dn
Ts65Dn
Ts65Dn
Tubulin
-50

For S1A Fig

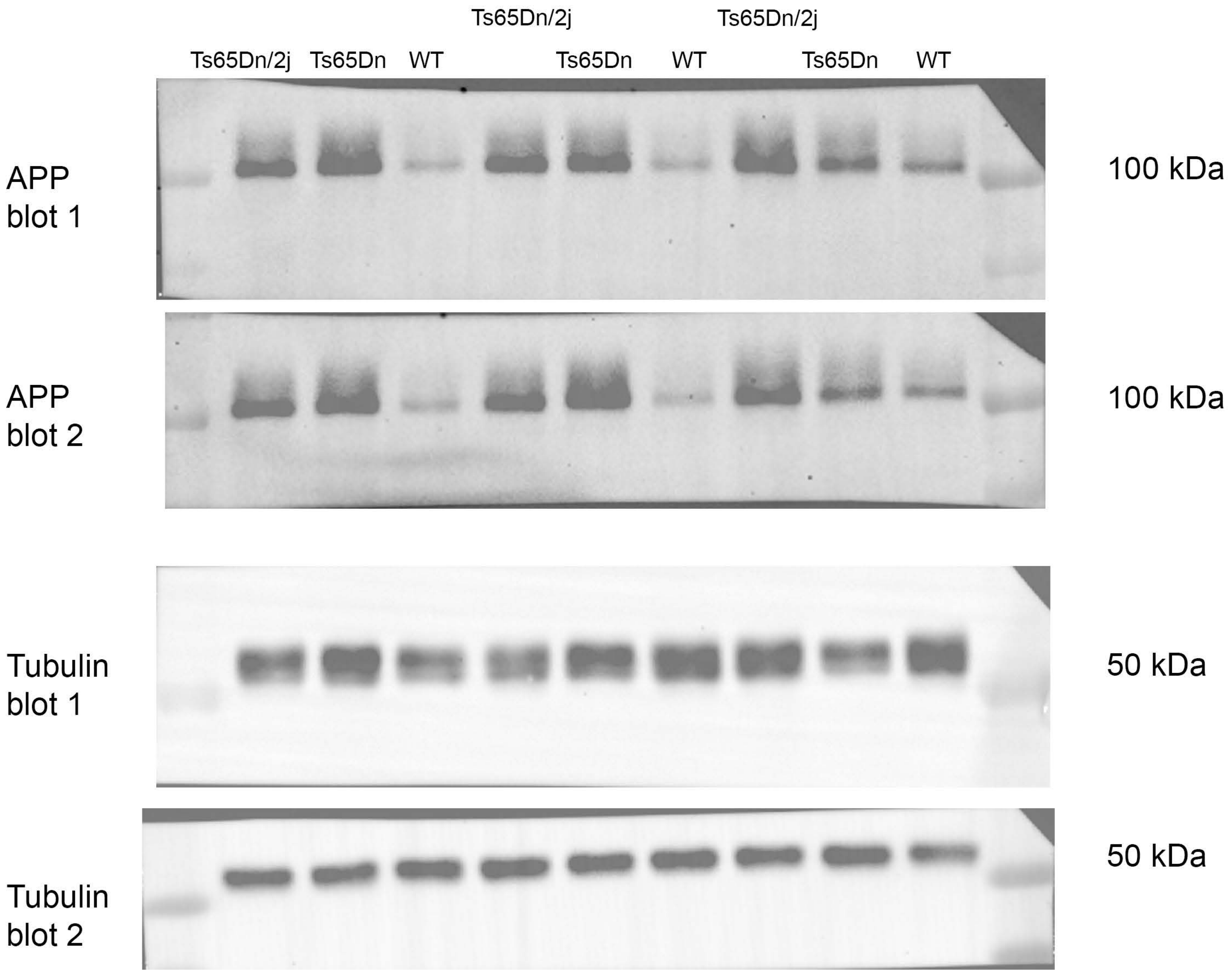
Ts65Dn/2j
Ts65Dn
WT
Ts65Dn/2j
Ts65Dn
WT
Ts65Dn/2j
Ts65Dn
WT
APP blot 1
100 kDa
APP blot 2
100 kDa
Tubulin blot 1
50 kDa
Tubulin blot 2
50 kDa

For S8A Fig

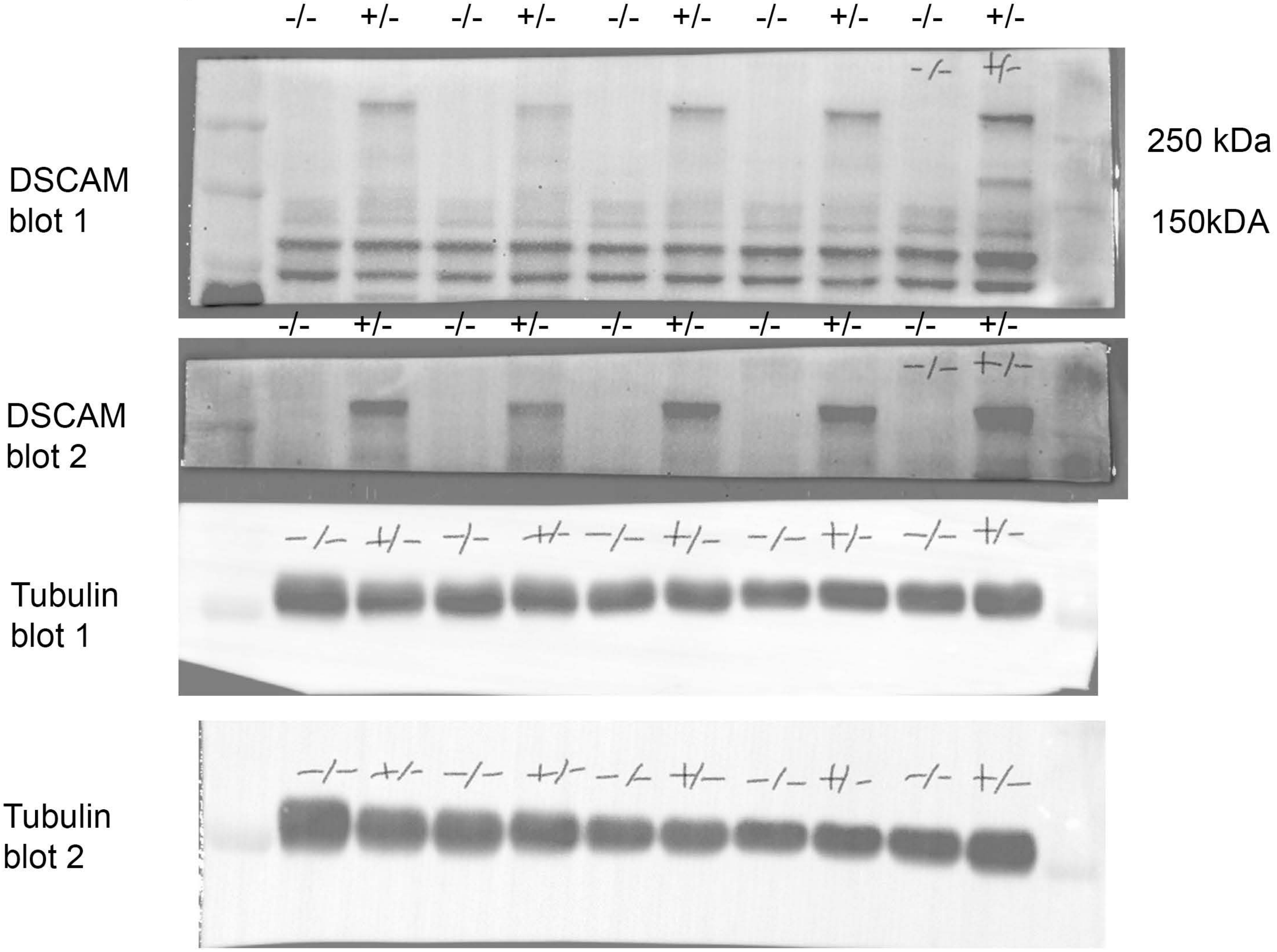

-/- +/- -/- +/- -/- +/- -/- +/- -/- +/-
DSCAM blot 1
250 kDa
150kDA
-/- +/- -/- +/- -/- +/- -/- +/- -/- +/-
DSCAM blot 2
Tubulin blot 1
Tubulin blot 2

For S8B Fig

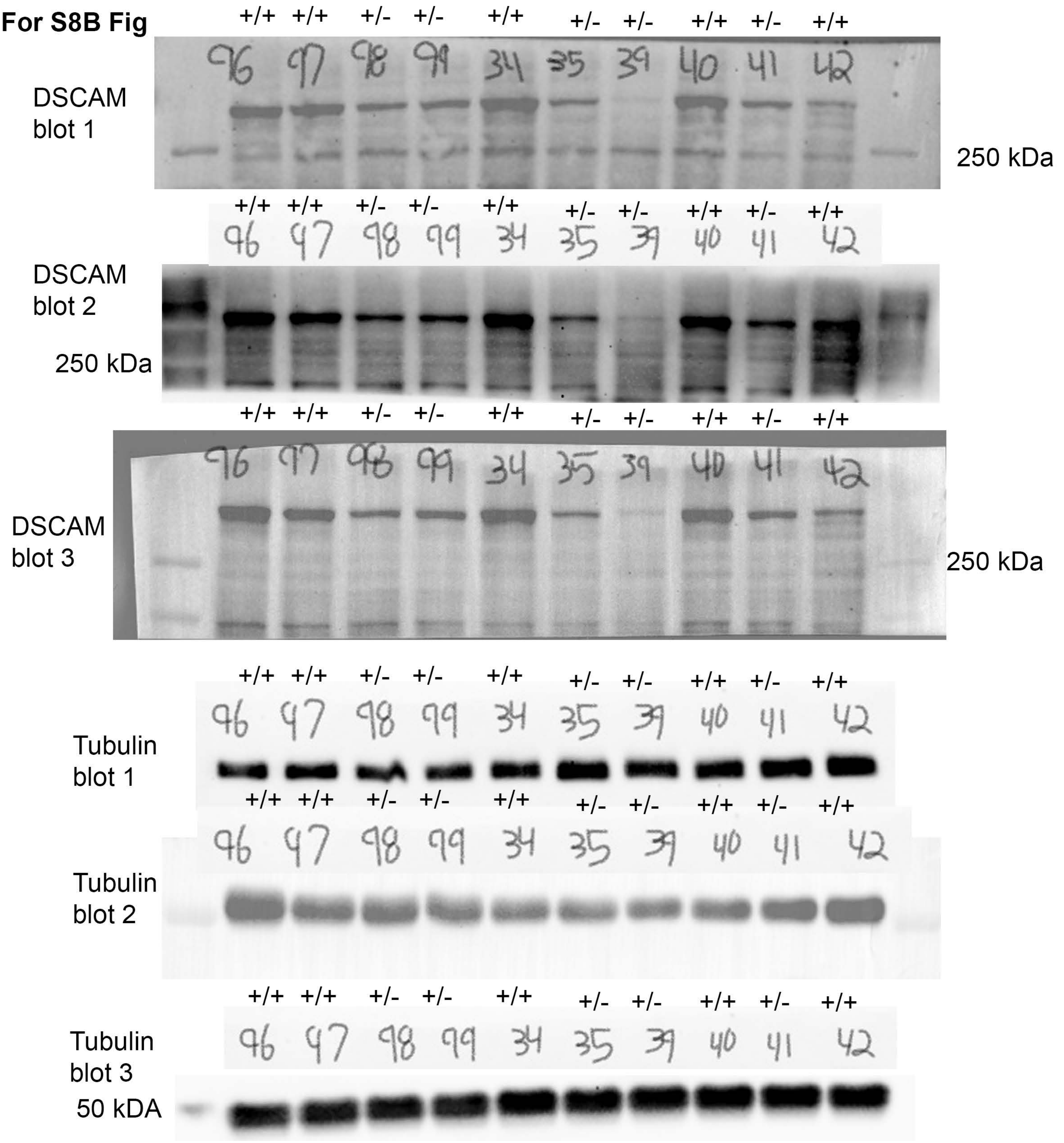
+/+ +/+ +/- +/- +/+ +/- +/- +/+ +/- +/+
DSCAM blot 1
250 kDa
DSCAM blot 2
250 kDa
DSCAM blot 3
250 kDa
Tubulin blot 1
Tubulin blot 2
Tubulin blot 3
50 kDA

For S10 Fig

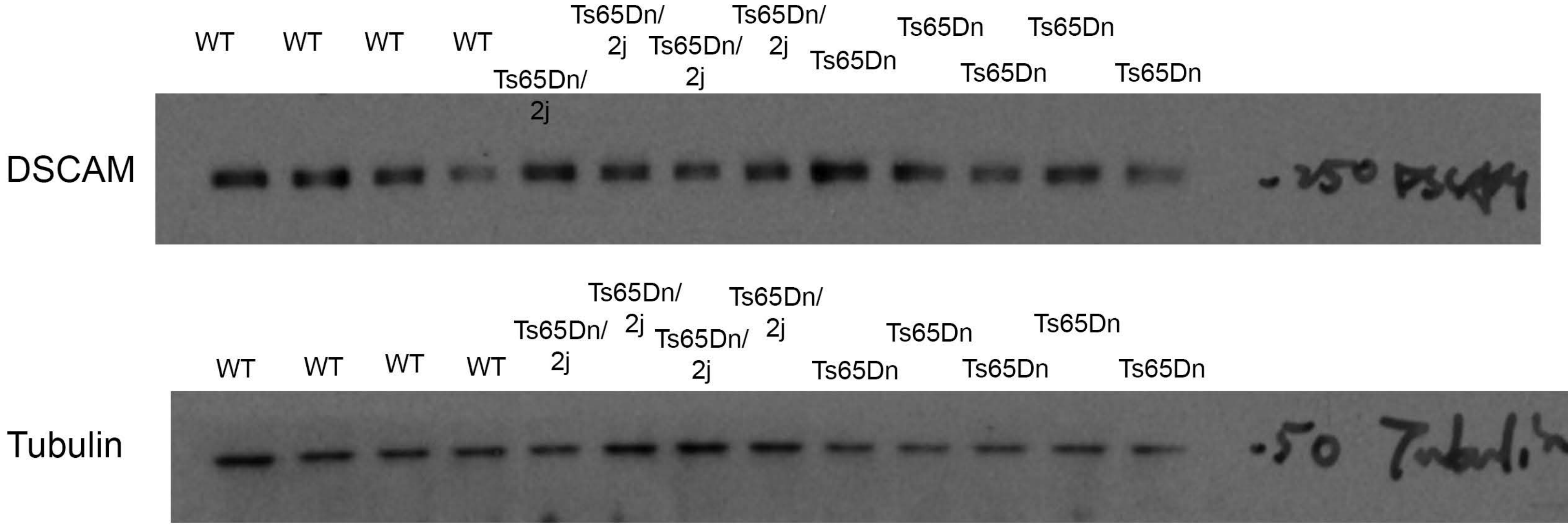
WT
WT
WT
WT
Ts65Dn/ 2j
Ts65Dn/ 2j
Ts65Dn/ 2j
Ts65Dn/ 2j
Ts65Dn
Ts65Dn
Ts65Dn
Ts65Dn
Ts65Dn
DSCAM
WT
WT
WT
WT
Ts65Dn/ 2j
Ts65Dn/ 2j
Ts65Dn/ 2j
Ts65Dn/ 2j
Ts65Dn
Ts65Dn
Ts65Dn
Ts65Dn
Ts65Dn
Tubulin
