# Supplementary material for: DSCAM gene triplication causes excessive GABAergic synapses in the neocortex in Down syndrome mouse models
Source: PLoS Biol. 2023 Apr 20;21(4):e3002078. doi: 10.1371/journal.pbio.3002078 (PMC10118173; doi:10.1371/journal.pbio.3002078)
Supplement: Attachment — Submitted filename: Ye_point-by-point response.pdf [file pbio.3002078.s013.pdf]

I. Major changes to the manuscript

We are encouraged by the positive feedback from all four reviewers, and very much appreciate their constructive critiques. Despite the limitations on laboratory activities imposed by COVID-19 since mid-March, 2020, we have worked hard to address the reviewers' concerns and improved the paper. We performed many additional experiments and extensively revised the manuscript. The following is a list of major new changes added to the revised manuscript. We have also made a number of efforts to improve the presentation.

We restructured the paper by (1) focusing on the Down syndrome mouse model; and (2) strengthening the studies on the GABAergic synapses that basket cells form on the somas of cortical pyramidal neurons (PyN) and present these studies before presenting ChC axon terminal data. The new results support a link between the morphological and electrophysiological data (Reviewer 2&3's comments). They also suggest that DSCAM's role in altering GABAergic synapse development in the trisomic mice is a more general process.

The major new results added to the revised manuscripts are:

1. We determined whether an extra copy of *DSCAM* gene <u>in GABAergic neurons</u> caused the excessive GABAergic synapses to form on the soma of PyNs. This was achieved by selectively normalizing *DSCAM* gene dosage in GABAergic neurons through integrating a Cre mouse line and a floxed *DSCAM* allele in the trisomy mice. We examined both the morphological synapses (new Figure 1D&F) and the synaptic currents (new Figure 2D-F and S3D-F). The results are consistent with those obtained by normalizing *DSCAM* gene dosage in the entire animal, and suggest that the extra copy of *DSCAM* gene in GABAergic neurons leads to an excessive number of GABAergic synapses and an increase in inhibitory postsynaptic currents on PyN somas.

2. We show that loss of *DSCAM* impairs the perisomatic GABAergic synapses innervating PyNs (Figure 4A-D).

3. We found that the soma size of PyNs in the ACC is not affected by the Ts65Dn or Ts65Dn:*DSCAM*+/+/- genetic background (Figure S1D). We also quantified the AIS length of PyNs in layer II/III of the ACC and found that they were not affected by either trisomy or normalizing DSCAM in the trisomic background (Figure S7B)

4. We show that genetic normalization of *DSCAM* gene dosage rescues the excessive GABAergic synapses formed on the PyN somas in the somatosensory cortex in Ts65Dn mice (Figure S2), which suggests that *DSCAM*'s contribution to the development of GABAergic synapse in Ts65Dn is beyond the anterior cingulate cortex (ACC).

5. We characterized the excitability, firing rate, single action potential and cell membrane properties, as well as sEPSCs of PyNs and ChCs (Figure S4 and S11).

6. We quantified DSCAM levels in *DSCAM*$^{+/+}$ (+/+), *DSCAM*$^{2j/+}$ (+/-), and *DSCAM*$^{2j/2j}$ (-/-) mice (Figure S8).

To present the work more clearly, we re-arranged some figure panels that either might cause confusion or are inaccurate according to the reviewers' comments. These changes do not affect the conclusions. We also changed some wording and terminologies to improve the readability of the manuscript. Moreover, we added more descriptions of the methodology as well as explanations of the rationales in the Results section, as suggested by the reviewers. Furthermore, we have made major revisions to the Discussion section based on the reviewers' comments to extend the depth of the discussion and make it more comprehensive.

II. Point by point responses

We would like to thank the reviewers for their thoughtful and constructive comments. By addressing their concerns, we have strengthened the paper. The following are our responses to reviewers' comments; major changes in the main text are in blue.

Reviewer #1

Major Comments:

1. In this reviewer's view, overall conceptual advance and rationale is not sufficient. For example, why did the authors focus on neocortex for this study? Is there any strong evidence that Down syndrome pathogenesis is mainly involved in synaptic/circuit dysfunctions in neocortical neurons?

> We have added a paragraph in the Introduction to explain why we focused on the neocortex (ln 77-89). Briefly, in contrast to the large body of literature on GABAergic signaling in the hippocampus of DS animal models, very little is known about whether GABAergic signaling is altered in the neocortex. Previous studies showed that the sizes of synaptic boutons and inhibitory synapses are enlarged in the neocortex of Ts65Dn mice, suggesting possible alterations in GABAergic synaptic functions in the neocortex (Belichenko et al., 2009; Belichenko et al., 2004; Kurt et al., 2000). We demonstrate that GABAergic inhibition of neocortical PyNs is excessive in Ts65Dn mice and that DSCAM overexpression plays a key role in this process.

2. The authors also should characterize the loss-of-function effects of other GABAergic interneurons beyond chandelier cells (e.g. parvalbumin-positive cells).

> In the revised manuscript, we add the loss-of function study of the perisomatic GABAergic synapses formed by basket cells, the other major type of parvalbumin-positive neurons in neocortex. Our results show that loss of *DSCAM* reduces the number of GABAergic synapses on PyN somas (new Figure 4).

3. In some of the experiments, the authors used heterozygous mice as a control. WT littermate mice should be in parallel analyses.

> This comment is about the loss-of-function experiments (- all the experiments involving trisomy models included wild-type littermates). In the initial submission, we compared only heterozygous and homozygous mice for a technical reason. *DSCAM*$^{2j/2j}$ (-/-) pups are weak. To promote their survival we euthanized wild-type littermates at P5-7. It is thus almost impossible to collect wild-type, heterozygotes, and homozygotes from the same litter. (An explanation of this has been added to the Methods, ln 517-521.) In the revised manuscript, we examined perisomatic GABAergic synapses in *DSCAM*$^{+/+}$ (+/+) and *DSCAM*$^{+/2j}$ (+/-) mice (new Figure 4C-D). Compared to wild-type littermates, *DSCAM*$^{+/-}$ showed a mild reduction in their GABAergic perisomatic synapses. As DSCAM protein level was reduced by 19% in *DSCAM*+/- heterozygotes (new Figure S8B), these results are consistent with a dose-dependent role for DSCAM in synaptic development (Kim et al., 2013). It is thus essential to compare the heterozygotes with homozygotes. As we showed, *DSCAM*$^{2j/2j}$ (-/-) homozygotes displayed further reduction in perisomatic GABAergic synapses (new Figure 4A-B).

4. Mechanistic understanding is lacking why normalization of Dscam level rescued the cellular phenotypes of Ts65Dn mice. Given Dscam binds to itself and other synaptic membrane proteins, it

would be essential to show that known molecular action of Dscam contributes to the maintenance of appropriate GABAergic innervation and inhibition. This experiment is important because it is meaningful to support the title of this manuscript.

> It will be important to identify both upstream and downstream signaling events related to DSCAM's role in neural development, but we believe that detailed molecular mechanism is beyond of the scope of this paper. In this paper, we demonstrate causality by restoring DSCAM level in trisomy background to that of wild-type and show a rescue of synaptic and morphology defects. The trisomy mice are unfortunately poor breeders. It took us 2-3 years to get enough mice for statistical analyses in the initial submission and over two years in revision. The major contribution of this paper is the demonstration that DSCAM overexpression is responsible for the excessive GABAergic synapses in the neocortex in Down syndrome model mice, rather than the biological role of DSCAM in GABAergic synapse development. To reflect this major contribution, we have revised the manuscript so that it focuses on the role of DSCAM in Down syndrome mouse model.

To further address the reviewer's concern, in the revised manuscript we include a new study (again a highly challenging experiment due to the complex genetics and difficulty in breeding) to determine whether overexpressed DSCAM functions in GABAergic neurons to cause the changes in GABAergic synapses. This study, which includes both morphological and electrophysiological experiments, required conditional knockout of one copy of the *DSCAM* gene in the trisomic genetic background. The results suggest that the extra copy of *DSCAM* gene in GABAergic neurons leads to the excessive GABAergic synapses on PyN somas (new Figure 1D&F, 2D-F and S3D-F). We believe that this important experiment moves a significant step further to elucidating the molecular mechanism.

5. For immunohistochemistry experiments, number of mice, not number of brain slices, should be used for statistical analyses.

> In the revised manuscript, we changed the samples to mice in all immunohistochemistry experiments and performed statistical analysis again (new Figure 1E-F, 3C/E/F, 4B/D, 5B/D/E, S1D, S2B, S5B/D, and S7).

6. For electrophysiology experiments in Figures 2 and 6: alterations of presynaptic chandelier neurons should be analyzed (e.g. excitability). Moreover, measurement of mEPSCs (or sEPSCs) and paired-pulse ratio should be analyzed to support the authors' claim.

> We thank the reviewer for pointing this out. In the revised manuscript, we include results of the excitability, firing rate, and EPSCs of PyNs and ChCs (Figure S4 and S11). This experiment required time-consuming genetic crosses to generate the mice.

As we discussed with the editors, paired-pulse ratio (PPR) is beyond the scope of this paper. Technically, measuring PPR between chandelier cells and pyramidal cells is very challenging, if possible at all, since they form GABAergic synapses on the initial segments of pyramidal cells. Moreover, PPR analysis is more appropriate for detecting changes in neurotransmitter release probability than for the number of synapses, and requires applying stimulations that causes depolarization. Our paper reports how GABAergic synapses are altered by DSCAM overexpression in trisomy associated with Down syndrome; as Reviewer 3 commented, "*the bouton number is increased which is consistent with an increase in the number of inputs (mIPSCs)*". Thus, we think mechanisms involving the release probability, which might be shown by PPR analysis, are interesting but not core to the subject.

7. It would strengthen this manuscript if authors could perform a subset of behavior analyses testing core features of Down syndrome model mice, and analyze whether normalization of Dscam levels could rescue the behavioral deficits.

> As the editors pointed out in the decision letter, this is out of the scope of this paper.

Minor Comments:

1. Title is overstated - this study did not clearly demonstrate causality of Dscam gene triplication and overinhibition in neocortical circuits.

> We believe we did demonstrate causality by restoring *DSCAM* dosage in trisomy background to that of wild-type. In the revised manuscript, we further identified the cell-type specificity of this effect by selective normalization of *DSCAM* dosage in GABAergic neurons.

2. Various typographical errors should be corrected: line 29 (contributes), line 93 (wild-type), line 898 (delete one of 'percentage').

> We have corrected the typos.

3. Line 135 - subtitle should be consistently in present tense.

> Corrected.

4. Figure 1B: it appears that Dscam KO neurons exhibit more cartridge numbers, contrary to the data presented in Figure 1C.

> The original Figure 1B&C are now Figure 5A&B. The images are representative. Besides a slight reduction in the cartridge length, axon cartridges of ChCs in *DSCAM-/-* appeared to project horizontally more often than *DSCAM+/-*, which might have caused the perception that *DSCAM-/-* exhibit more cartridges than *DSCAM+/-*.

5. I am not convinced whether anti-phospho-IkB is a reliable marker for AIS - the authors should verify their claim using multiple markers.

> We have included a result for quantitative comparison of the percentage of AIS labelled by anti-AnkG (another AIS marker) and/or anti-phospho-IkB (new Figure S6B-C). The result shows that AIS labeling by these two antibodies are very close to each other. Anti-phospho-IkB is commonly used as an AIS marker (Fazzari et al., 2010; Lu et al., 2017; Tai et al., 2014; Taniguchi et al., 2013).

6. Figure 2G-H: an increase in c-Fos positive neurons is not sufficient to demonstrate the title of Figure 2. In addition, did authors observe any changes in intensity of c-Fos immunoreactivity?

> We have removed the c-Fos staining data from the manuscript.

7. Figure 6: Representative traces in Figure 6E and H do not match with summary results presented in Figure 6G and J.

> The reviewer probably referred to the amplitudes of IPSCs in those figures (now Figure 2A&C). As the quantifications show, the amplitudes of the IPSCs are highly variable. Thus, some IPSCs with large amplitudes are seen in the traces, possibly giving the impression that the mean amplitude are larger.

8. A decrease in parvalbumin-positive neurons in ACC of Ts65Dn mice is surprising and puzzling. Authors should provide reasonable explanation why this occurs, and examine whether other interneurons are altered.

> We showed an increase, instead of decrease, in parvalbumin-positive neurons in ACC of Ts65Dn mice (original Figure S7A-B; new Figure S5A-B). This is consistent with a previous report (Chakrabarti et al., 2010). Notably, normalizing DSCAM levels did not rescue the increased density of PV+ neurons in the ACC region of Ts65Dn mice (new Figures S5A-B), but rescued the increased number of perisomatic GABAergic synapses on PyNs. These results are consistent with the notion that the overexpressed DSCAM in the Ts65Dn neocortex causes excessive GABAergic synaptic transmission by increasing the number of GABAergic synapses.

Reviewer #2

Major concerns:

1. The data nicely support the idea that Dscam levels are a regulator of inhibitory synaptic innervation. However, is this a direct or indirect relationship? Might alterations in neuronal activity or other synaptic inputs lead to secondary disruption of inhibitory synaptic formation or maintenance? If it is direct, could the authors speculate on molecular pathways that may participate (synaptic scaffolding, trafficking, plasticity)? Additionally, there is no evidence for whether Dscam acts cell autonomously (or even whether it is acting pre- or postsynaptically). The authors note some experimental challenges to this problem, such as sparsely removing Dscam from chandelier cells. However, they appear to assume that all Dscam actions are presynaptic. If Dscam acts postsynaptically, it is straightforward to sparsely delete the gene from pyramidal cells (e.g., via low-titer viral cre expression) and examine the consequences for inhibitory innervation.

> In the revised manuscript, we have made extensive effort to address the reviewer's question whether *DSCAM* acts cell autonomously (i.e., whether it is acting pre- or post-synaptically). We include a new study (- a highly challenging experiment due to the complex genetics and difficulty in breeding) to determine whether overexpressed DSCAM functions in GABAergic neurons to cause the changes in GABAergic synapses. This study, which includes both morphological and electrophysiological experiments, required conditional knockout of one copy of the *DSCAM* gene in the trisomic genetic background. The results suggest that the extra copy of *DSCAM* gene in GABAergic neurons leads to the excessive GABAergic synapses on PyN somas (new Figure 1D&F, 2D-F and S3D-F). We believe that this important experiment move a significant step further to support the idea that DSCAM levels act in GABAergic neurons (i.e., presynaptically) to affect GABAergic synapse development in trisomy mice.

Moreover, we show in the revised manuscript that the excitability of PyN and ChC are largely unaffected in the trisomy mice or those with DSCAM dosage corrected (new Figure S4 &S11). These results suggest that it is unlikely that neuronal activity is responsible for the morphological and synaptic changes that we observed. Instead, they further supports that DSCAM's action is direct. We have added a discussion on the role of neuronal activity in the formation and maintenance of inhibitory synapses (ln 429-430). In the revised Discussion, we also speculate the molecular pathways (ln 426-429).

We believe that the major contribution of this paper is the demonstration that DSCAM overexpression is responsible for the excessive GABAergic synapses in the neocortex in Down syndrome models. As the molecular basis of the excessive GABAergic inhibition in the neocortex of Down syndrome models is unknown, we believe this is an important report in the field of Down syndrome research. How DSCAM overexpression causes the excessive GABAergic innervation in the neocortex requires much more studies to complete. The trisomy mice are unfortunately poor breeders. It took us 2-3 years to get enough mouse numbers for statistical analyses in the initial submission and over two years in revision.

2. The role of chandelier cells in the synaptic deficits identified are unclear. Indeed, the data show that perisomatic inhibitory synapses are also altered, suggesting a general process of weakened inhibition. Are dendritic inhibitory inputs also disrupted? Overall, there are no data directly supporting links between changes in chandelier axons and synaptic changes, making the morphological and electrophysiological data very disjointed in the manuscript.

> We thank the reviewer for this insightful comment. Indeed, the synaptic responses in PyN are unlikely to be from ChCs. As Reviewer 3 pointed out, the parvalbumin basket cells are the major population of

interneurons in the cerebral cortex. Considering the reviewers' comments, the revised manuscript (including new results) focuses on perisomatic GABAergic synapses, which are formed by the parvalbumin basket cells, and present the chandelier cell studies in a less prominent way. These new results further suggest a general role of *DSCAM* in the changes in GABAergic synapse development in Down syndrome mouse models and support a link between the morphological and electrophysiological data.

3. Almost all statistics are carried out using "cell" or "field of view" for degrees of freedom. However, cells and fields of view in the same animal are not independent, violating basic statistical assumptions. All analyses (Fig. 1 C,E,G, Fig. 2 B-H, etc.) should be re-done using by-animal analyses. Additionally, p-values are not presented in any figures or legends after Figure 1 - please add these values to the manuscript. Finally, in the Figure 1 legend, I do not understand what it means to use a "one-way ANOVA followed by t-test". Please clarify.

> In the revised manuscript, we changed the samples to mice in all immunohistochemistry experiments and performed statistical analysis again (new Figure 1E-F, 3C/E/F, 4B/D, 5B/D/E, S1D, S2B, S5B/D, and S7).

We have added P-values to the legend for each figure in the revised manuscript. Moreover, we changed the phrase "*one-way ANOVA followed by t-test*" to "one-way ANOVA for multi-group comparisons and *post hoc* Student's *t*-tests for pair-wise comparisons" (in Methods and Figure legends).

Reviewer #3

This is an elegant study with some interesting findings, however I have some major concerns that the authors need to address before considering its publication in PLoS Biology.

1) The argument on why chandelier cells were chosen to explore the role of Dscam in inhibition is a bit weak. The parvalbumin basket cells are the major population of interneurons in the cerebral cortex, and even though chandelier cells target many pyramidal cells whether they provide the most potent inhibition that pyramids receive or what does it means, it is still under debate. Is this cell adhesion molecule particularly express or enriched in chandelier cells versus other neurons?

> We thank the reviewer for this insightful comment. Indeed, we had no reason to believe that DSCAM is particularly expressed or enriched in chandelier cells. We used ChCs to study the morphological changes in their axon terminals due to the availability of the genetic labeling technique and the relative stereotyped morphology of ChCs – as compared to other types of GABAergic neurons. Importantly, because ChCs innervate PyNs at AIS – the spike initiation site, they are thought to exert the most powerful inhibition that controls the output of PyNs. The electrophysiological phenotypes that we observed in PyN are also unlikely to be from ChCs. Considering these, the revised manuscript focuses on perisomatic GABAergic synapses, which are formed by the parvalbumin basket cells, and present the chandelier cell studies in a less prominent way. We also specifically corrected *DSCAM* gene dosage in cortical GABAergic neurons and found that the extra copy of *DSCAM* gene in GABAergic neurons leads to the excessive perisomatic GABAergic synapses on PyNs. These new results further suggest a general role of *DSCAM* in the changes in GABAergic synapse development in Down syndrome mouse models.

We have deleted the sentence "ChCs are thought to be the most potent inhibitory neurons in the neocortex".

What does it mean that: "ChC cartridge length and synaptogenesis are proportional to neocortical Dscam expression levels"? Discussion page 13. Please clarify this.

> In the revised Discussion, we have changed the sentence to "Notably, ChC cartridge length, bouton number and bouton sizes correlate with DSCAM expression levels (Figures S10B-D), suggesting the sensitivity of ChC development to DSCAM levels".

Although it is not a proof of protein expression, there are several databases illustrating the levels of transcripts in different cell types (scRNA and RNA bulk sequencing). It would be nice to have this data in the manuscript and/or some proof that Chandelier cells express Dscam during development and/or in adulthood.

> scRNA studies show that ChC and BC express *DSCAM* (Paul et al., 2017).

2) Whereas is clear that Ts65Dn model show an increase in Dscam protein levels by western blot, the authors do not provide any evidence of no protein level changes in the Dscam mutants and hets compare to controls. This is particularly relevant, since the authors use Dscam+/- as control in Figures 1 and 2. Are the Dscam+/- identical to wild-type in the number of synapses? It would be important that the authors provide some kind of proof that there is no phenotype with only one copy of Dscam (Dscam+/-).

> This comment is about the loss-of-function experiments; we included wild-type littermates in all experiments involving trisomy models. In the initial submission, we compared only heterozygous and homozygous mice for a technical reason. *DSCAM*$^{2j/2j}$ (-/-) pups are weak, so to promote their survival we euthanized wild-type littermates at P5-7. It is thus almost impossible to collect wild-type, heterozygotes and homozygotes from the same litter. (An explanation of this has been added to the Methods, ln 517-521). In the revised manuscript, we examined perisomatic GABAergic synapses in *DSCAM*$^{+/+}$ (+/+) and *DSCAM*$^{+/2j}$ (+/-) mice (new Figure 4C-D). Compared to wild-type littermates, *DSCAM*+/- heterozygotes showed a mild reduction in their GABAergic perisomatic synapses. Moreover, the DSCAM protein level was reduced by 19% (new Figure S8B). These results are consistent with the notion that DSCAM function in synaptic development is dose-dependent (Kim et al., 2013). It is thus essential to compare the heterozygotes with homozygotes. As we showed, *DSCAM*$^{2j/2j}$ (-/-) homozygotes displayed further reduction in perisomatic GABAergic synapses (new Figure 4A-B).

3) The authors claim that, a change in mIPSC or sIPSC are due to a reduction in chandelier boutons. This is misleading since patch clamp recordings in pyramidal cell are taking events from the soma (basket cells) as well as from the AIS (chandelier). To my knowledge, it is not possible to distinguish the contribution of each population. Then, as it is now the paper, shows some inconsistency:
-Figure 1 and 2. They explore the function of Dscam using the Dscam mutants and show changes in presynaptic terminals (cellular) in chandelier and synapses (chandelier and/or basket cells, electrophysiology).
-Figure 3. They show changes in presynaptic terminals (cellular) in chandelier in the Ts65Dn mouse model.
-Figure 4 and 6. They show the rescue of presynaptic terminals (cellular) and synaptic (electrophysiology) in both chandelier and basket cells of the Ts65Dn model when they reduce the doses of Dscam by including a mutant Dscam allele (Dscam het).

> The reviewer is correct that patch clamp recordings in pyramidal cell do not distinguish the events taking place from the soma (postsynaptic to basket cells) or the AIS (postsynaptic to chandelier cells). As discussed in our response to the reviewer's comment #1, because parvalbumin basket cells are the major population of interneurons in the neocortex, the revised manuscript focuses on perisomatic GABAergic synapses, which are formed by the parvalbumin basket cells. We also corrected *DSCAM* gene dosage in cortical GABAergic neurons and found that the extra copy of *DSCAM* gene in GABAergic neurons leads to the excessive perisomatic GABAergic synapses on PyNs. These new results further suggest a general role of *DSCAM* in the changes in GABAergic synapse development in Down syndrome mouse models and support a link between the morphological and electrophysiological data.

I am missing in Figure 1 and Figure 3 the cellular presynaptic analysis in basket cells, as they did in Figure 6. By doing this, the authors do not need to artificially claim that the changes in mIPSCs frequency is due to a deficit in chandelier cells or that they particularly expressed DSCAM. I think this, together with the elegant experiments in Figure 4 and 6, will strengthen the manuscript, showing that Dscam function is relevant for inhibitory synapse formation, and the excess of Dscam contributes to the synaptic phenotypes found in the Ts65Dn model.

> The reviewer's comment refers to the missing analysis in basket cells in the loss-of-function studies of *DSCAM* (+/- vs -/-) in the original Figures 1 and 3. In the revised manuscript, we have added this result in the new Figure 4A-B. We also added the comparison between *DSCAM*$^{+/+}$ and *DSCAM*$^{+/-}$ (new Figure 4C-D). Compared to wild-type littermates, *DSCAM*+/- heterozygotes showed a mild reduction in their

GABAergic perisomatic synapses. As the DSCAM protein level was reduced by 19% in *DSCAM*+/- heterozygotes (new Figure S8B), these results are consistent with the notion that DSCAM function in synaptic development is dose-dependent (Kim et al., 2013).

4) Does the AIS or soma size in pyramidal cells change in the Dscam mutant or Ts65Dn model? The AIS length and the soma are different among pyramids, and also the AIS length change with activity. Since the authors quantify per chandelier cells this is an important issue to address. For example, if Ts65Dn model shows an increase in AIS size, the change in the bouton number could be secondary to a change in the AIS in pyramidal cells that also lack Dscam.

> We have added quantifications of AIS and soma sizes in the revised manuscript (Figure S1D and S7B).

5) The mIPSCs frequency changes in both (Dscam mutants-loss of function) and Ts65Dn model (Dscam gain of function). However, the amplitude is only altered in the Dscam mutants. Why the post-synaptic response is not Dscam dose-dependent? The authors need to discuss this.

> We have added a discussion as suggested (ln 357-368).

6) The authors claim: "we found a strong correlation between the cartridge and the bouton number of each ChC in both wild-type and Ts65Dn mice" "There was also a significant correlation between cartridge length and bouton size in both wild-type and Ts65Dn mice (Figure 5C)".

Considering the two positive correlations, it is difficult to exclude that changes in expression of Dscam can influence cartridge's length, which consequently may secondary affects bouton number and size. For example, in figure 5A, with a 100 microns cartridge length, the number of presynaptic boutons is the same for euploid and Ts65Dn genetic background. Nevertheless, I think that although these correlations are interesting, they did not provide too much new information to go as principal figure. I would suggest passing Figure 5 to supplementary information.

> We have moved the original Figure 5 to the supplementary information (new Figure S9).

7) Figure 2, c-fos staining is not a reliable proof of increase in activity. Why the authors did not measure instead the spontaneous EPSCs in the pyramidal cells?

> We have removed the c-Fos result from the manuscript.

8) The data used for Figures 3B- D and 4D-F for the Euploid and Ts65Dn samples are the same. This is fine, but it would be good that the authors mention this in the Figure legends to avoid misunderstandings.

> We have combined these figures into one (new Figure 3). As a result, the data are not presented in two different figures any more.

Minor points

- Figure S7, the authors should show a bigger area to illustrate parvalbumin density instead of a crop image.

> We show a larger area in the revised Supplementary Information (new Figure S5).

-Page 11: "we found that mIPSC frequency was increased by ~63% in Ts65Dn mice compared to euploid littermates (Figures 6E-F). These results suggest an increase in presynaptic neurotransmitter release". This statement is not correct. mIPSC frequency provides information about the number of inputs and the presynaptic release sites. The authors have shown that the bouton number is increased which is consistent with an increase in the number of inputs (mIPSCs). A potential presynaptic release increase can only be tested by assessing the paired pulse ratio (PPR), an experiment that the authors did not perform.

> We thank the reviewer for pointing this out. We have changed the sentence to "We found that mIPSC frequency was increased by ~63% in Ts65Dn mice compared to euploid littermates (Figures 2A and 2B), which is consistent with enhanced synaptogenesis in basket cells (Figures 1C and 1E)" (ln 167-170).

Reviewer #4
Many thorough and effective methods were used in this paper that increase its merit. These include double-blinding the researchers during data analysis and imaging. There are a number of questions/issues, however, which in my opinion would significantly strengthen the study, if addressed.

Controls
- Can the authors confirm that DSCAM levels are reduced in the Dscam -/- mouse ACC as they do for the other mouse lines (e.g., Ts65Dn)?

> We have added western blotting results that compared the DSCAM levels in *DSCAM-/-* and *DSCAM+/-* as well as *DSCAM+/+* with *DSCAM+/-* (New Figure S8). As reported previously (Fuerst et al., 2010; Schramm et al., 2012), DSCAM protein was not detected in *DSCAM*$^{-/-}$ by western blotting.

- In Fig S5A, one of the four Ts65Dn++- mice with expected normalised DSCAM levels, has in fact higher/similar DScam levels than three out of five of the Ts65Dn mice. In which plots/statistical analyses were the data from this mouse used and have the results been discussed?

> Fig S5 is now Fig S10 in the revised manuscript. Figure S5B-D (now Figure S10B-D) show that this special Ts65Dn:*DSCAM*+/+/- mice shows longer axons, more bouton numbers than that of other 3 Ts65Dn: *DSCAM* +/+/- mice, supporting the notion that DSCAM levels determine ChC presynaptic growth.
- It would be useful to check that other Ts65Dn triplicated genes other than DSCAM have not been normalised during breeding, confounding the conclusions from the DSCAM level rescue experiments with Ts65Dn++- mice.

> We checked the level of APP (Amyloid Precursor Protein), which is encoded by another HSA21 gene that is important for brain disorders of DS, and found that normalizing *DSCAM* did not change APP protein levels (new Figure S1A).

Dscam function
- A powerful model to dissect the function of DSCAM in inhibitory synapse maturation would be to overexpress/delete DSCAM specifically in ChCs, basket cells or both and then study the effects on the development of inhibitory synapses and the AIS. This would further strengthen the conclusions of this study and provide new insights into the role of Dscam in inhibitory terminal maturation, without the confounding genetic variability of the Ts65Dn mouse model, where numerous other genes are overexpressed/altered.

> To further address the reviewer's concern, in the revised manuscript we include a new study (a challenging experiment due to the complex genetics and difficulty in breeding) to determine whether overexpressed DSCAM functions in GABAergic neurons to cause the changes in GABAergic synapses. This study, which includes both morphological and electrophysiological experiments, required conditional knockout of one copy of the *DSCAM* gene in the trisomic genetic background. The results suggest that the extra copy of *DSCAM* gene in GABAergic neurons leads to the excessive GABAergic synapses on PyN somas (new Figure 1D&F, 2D-F and S3D-F). We believe that this important experiment move a significant step further to provide insights into how *DSCAM* dosage contribute to GABAergic synapse development in the Ts65Dn mouse model.

- *Can the ChCs cartridge structural defect in the Dscam -/- and Ts65Dn be due to a compensatory effect to defective pyr neuron AIS? Can the authors measure the AIS length in the mutants to exclude this possibility?*

> We have added quantifications of AIS length in the revised manuscript (Figure S7B & S7D). There is no difference among euploid, Ts65Dn, Ts65Dn:DSCAM+/+/- or between *DSCAM+/-* and *DSCAM-/-*. Moreover, to rule out the possibility that the changes in perisomatic synapses are due to changes in the size of PyN somas, we quantified PyN somas and found no difference among these genotypes (Figure S1D).

- *Does Dscam regulate ChCs numbers?*

> Although we do not have a way to label and thus count all ChCs, we show that PV+ neurons, which include both ChCs and PV+ basket cells (that form perisomatic synapses on PyNs) are not affected in *DSCAM*$^{2j/2j}$ (new Figure S5C-D), but increased in Ts65Dn (new Figure S5A-B). This is consistent with a previous report (Chakrabarti et al., 2010). Notably, normalizing DSCAM levels did not rescue the increased density of PV+ neurons in the ACC region of Ts65Dn mice (new Figures S5A-B), but rescued the increased number of perisomatic GABAergic synapses on PyNs. Thus, the overexpressed DSCAM in the Ts65Dn neocortex causes excessive GABAergic synaptic transmission by increasing the number of GABAergic synapses without affecting the number of PV+ GABAergic neurons.

- *What is the effect of Dscam over expression on Pyr neuron activity (e.g. excitability and firing rate) in ACC? How does the Dscam level normalisation affect Pyr neuron activity?*

> In the revised manuscript, we include results of the excitability, firing rate, and EPSCs of PyNs and ChCs (Figure S4 and S11).

- *Does Dscam regulate ChCs and basket cell axon length?*

> The vast majority of ChC axons are the axon cartridges that we quantified. Thus, our results suggest that DSCAM regulates ChC axon length. We used ChCs to study the morphological changes in their axon terminals due to the availability of the genetic labeling technique and the relative stereotyped morphology of ChCs – as compared to other types of GABAergic neurons. It is currently infeasible to study the morphology of basket cells at the resolution that we studied chandelier cells. This is due to the heterogeneity of basket cell morphology and the lack of genetic tools for labeling subtypes so that single-cell resolution can be achieved.

- *Does Dscam regulate also pyr neuron synaptogenesis or only inhibitory?*

> We believe that the major contribution of this paper is the demonstration that DSCAM overexpression is responsible for the excessive GABAergic synapses in the neocortex in Down syndrome models. As the molecular basis of the excessive GABAergic inhibition in the neocortex of Down syndrome models is unknown, we believe this is an important report in the field of Down syndrome research. It took us 2-3 years to get enough mouse numbers for statistical analyses in the initial submission and over two years in revision. We plan to study DSCAM's role in the development of excitatory synapses in the future.

- *The ACC is a relay between the pre-frontal and limbic systems, and may have a distinct role from other neocortical regions such as the visual, motor, and somatosensory cortices. Are the results*

presented expected to be valid for other cortical areas too. Some evidence or discussion should be included.

> To address the reviewer's question, we examined the somatosensory cortex. Our new result shows that genetic normalization of DSCAM levels rescues the excessive GABAergic synapses formed on the PyN somas in the somatosensory cortex in Ts65Dn mice (Figure S2). This result suggests that *DSCAM*'s contribution to in alterations in GABAergic synapse in Ts65Dn is beyond the ACC.

Minor point
What is the method of quantification used during GABAergic synapse analysis with Bassoon+ and VGAT+ puncta? The authors quantified places where Bassoon and VGAT signals were overlapping or apposed. If the signals were apposed, what is the range of distances allowed? How far apart did the signals need to be to still be considered part of the same synapse?

> We counted puncta that contained yellow pixels. Therefore, the correct description is "as indicated by Bassoon+ puncta that *overlapped with* VGAT+ signals" (ln 135). We have added a sentence describing this in the Methods section (ln 592-593).

References Cited in the Point-by-point Response

Belichenko, P.V., Kleschevnikov, A.M., Masliah, E., Wu, C., Takimoto-Kimura, R., Salehi, A., and Mobley, W.C. (2009). Excitatory-inhibitory relationship in the fascia dentata in the Ts65Dn mouse model of Down syndrome. The Journal of comparative neurology *512*, 453-466.

Belichenko, P.V., Masliah, E., Kleschevnikov, A.M., Villar, A.J., Epstein, C.J., Salehi, A., and Mobley, W.C. (2004). Synaptic structural abnormalities in the Ts65Dn mouse model of Down Syndrome. The Journal of comparative neurology *480*, 281-298.

Chakrabarti, L., Best, T.K., Cramer, N.P., Carney, R.S., Isaac, J.T., Galdzicki, Z., and Haydar, T.F. (2010). Olig1 and Olig2 triplication causes developmental brain defects in Down syndrome. Nat Neurosci *13*, 927-934.

Fazzari, P., Paternain, A.V., Valiente, M., Pla, R., Lujan, R., Lloyd, K., Lerma, J., Marin, O., and Rico, B. (2010). Control of cortical GABA circuitry development by Nrg1 and ErbB4 signalling. Nature *464*, 1376-1380.

Fuerst, P.G., Harris, B.S., Johnson, K.R., and Burgess, R.W. (2010). A novel null allele of mouse DSCAM survives to adulthood on an inbred C3H background with reduced phenotypic variability. Genesis *48*, 578-584.

Kim, J.H., Wang, X., Coolon, R., and Ye, B. (2013). Dscam expression levels determine presynaptic arbor sizes in Drosophila sensory neurons. Neuron *78*, 827-838.

Kurt, M.A., Davies, D.C., Kidd, M., Dierssen, M., and Florez, J. (2000). Synaptic deficit in the temporal cortex of partial trisomy 16 (Ts65Dn) mice. Brain Res *858*, 191-197.

Lu, J., Tucciarone, J., Padilla-Coreano, N., He, M., Gordon, J.A., and Huang, Z.J. (2017). Selective inhibitory control of pyramidal neuron ensembles and cortical subnetworks by chandelier cells. Nat Neurosci *20*, 1377-1383.

Paul, A., Crow, M., Raudales, R., He, M., Gillis, J., and Huang, Z.J. (2017). Transcriptional Architecture of Synaptic Communication Delineates GABAergic Neuron Identity. Cell *171*, 522-539 e520.

Schramm, R.D., Li, S., Harris, B.S., Rounds, R.P., Burgess, R.W., Ytreberg, F.M., and Fuerst, P.G. (2012). A novel mouse Dscam mutation inhibits localization and shedding of DSCAM. PLoS One *7*, e52652.

Tai, Y., Janas, J.A., Wang, C.L., and Van Aelst, L. (2014). Regulation of chandelier cell cartridge and bouton development via DOCK7-mediated ErbB4 activation. Cell Rep *6*, 254-263.

Taniguchi, H., Lu, J., and Huang, Z.J. (2013). The spatial and temporal origin of chandelier cells in mouse neocortex. Science (New York, NY *339*, 70-74.
