# Supplementary material for: DSCAM gene triplication causes excessive GABAergic synapses in the neocortex in Down syndrome mouse models
Source: PLoS Biol. 2023 Apr 20;21(4):e3002078. doi: 10.1371/journal.pbio.3002078 (PMC10118173; doi:10.1371/journal.pbio.3002078)
Supplement: Attachment — Submitted filename: 3_Point-by-point response.pdf [file pbio.3002078.s015.pdf]

Point-by-Point Response to Reviewers' Comments and Editorial Requests

We very much appreciate the reviewers and the editor's suggestions. In the revised manuscript that we are submitting, we have addressed the remaining minor concerns of the reviewers and the editorial requirements. Changes are highlighted in blue font color in both the main text and the Supporting Information document.

Reviewer #2: *The authors have done a nice job responding to my previous concerns. I have only one strong suggestion. I appreciate the authors' perspective that the proximal synapses are likely to be dominated by basket and chandelier cells (that are also likely to be PV-positive). However, there are some proximal inputs from other cell types (even SST-INs make some proximal synapses). Moreover, the Lhx6-Cre line will label PV- and SST-expressing cells. I suggest the authors temper their statements in several locations to note that "perisomatic" synapses seem to be altered and may be causal to the observed phenotypes. However, alteration of other inputs can't be ruled out. I don't think this change dampens the most important conclusions and places the overall study conclusions on more rigorous footing.*

> While the GABAergic synapses on the proximal dendrites of pyramidal neurons (PyNs) are not just formed by basket cells, those on the PyN soma are. In our studies of perisomatic synapses in this paper, we only quantified the GABAergic synapses (bouton, to be precise – see our response to Reviewer 3's comment below) on the PyN soma. Thus, our statement that the changes in the perisomatic boutons reflect those in basket cells is reasonable.

That being said, the reviewer is correct that the Lhx6-Cre line also labels SST-expressing cells, in addition to PV+ cells. To address this, we have added the following paragraph to emphasize that our results do not rule out the possibility that the alterations are caused by DSCAM in GABAergic neurons other than the basket cells (ln 338-346).

"Our studies using an *Lhx6*-Cre mouse line that targets GABAergic interneurons show that the extra copy of *DSCAM* in GABAergic neurons leads to the excessive GABAergic boutons on PyN somas (Figure 1D and F) and increased mIPSC frequencies in PyNs (Figure 2D-F). The *Lhx6*-Cre transgene is not only expressed in basket cells, which forms the perisomatic GABAergic synapses on PyNs [40, 41], but also expressed in chandelier cells and somatostatin+ GABAergic neurons [46, 47]. As such, these results do not demonstrate that the extra copy of *DSCAM* in basket cells causes the excessive boutons or increased mIPSC frequencies. Testing the cell-autonomous functions of *DSCAM* in perisomatic synapse development requires removing the extra copy of *DSCAM* specifically in basket cells."

Reviewer #3: *The authors have carried out a massive effort to address all of my concerns and most of the other reviewers' concerns. I do not have any further comments. The manuscript is solid and addresses a relevant question in the field which is not yet understood: the molecular mechanisms underlying the increase of inhibition in a Down Syndrome model.*

Minor
-Line 114, "…male loss-of-function" isn't it heterozygous instead of loss of function?

> We have changed this sentence to the following two sentences (Ln 104-107):

“Crossing female Ts65Dn mice with the male loss-of-function mutant of a gene of interest (e.g. *DSCAM*) yields trisomic mice with two functional copies of that gene. If heterozygous mutant is used, the progeny also includes the regular trisomic and euploid littermates (Figure 1B). “

-Since postsynaptic markers are not used (e.g. Gephyrin), it will be more appropriate to name them boutons or inputs, in the text and figures.

> We have changed “synapse(s)” to “bouton(s)” in all placed where the term refers to the presynaptic structures labeled by VGAT and Bassoon, including the figures, supplementary figures, and their legends. The places where we did not change the use of “synapse” are those supported by evidence. For example, “synapse” is used in the title because our electrophysiological experiments evaluate synaptic transmissions.

Other Editorial Requests

b) Please address my Data Policy requests below; specifically, we need you to supply the numerical values underlying Figs 1AEF, 2BCEF, 3CEF, 4BD, 5BDE, 6BCEF, S1AD, S2B, S3BCEF, S4ABCDEFGHIJ, S5BD, S7ABCD, S8AB, S9ABCDEF, S10BCDE, S11ABCDEFGHIJ, either as a supplementary data file or as a permanent DOI’d deposition.

> In the revised manuscript, we supply via a permanent DOI’d deposition the numerical values, blot images, and immunostaining images that underlie all figures, including the ones requested by the editor.

c) Please cite the location of the data clearly in all relevant main and supplementary Figure legends, e.g. “The data underlying this Figure can be found in S1 Data” or “The data underlying this Figure can be found in https://doi.org/XXXX” https://zenodo.org/record/7554842

> The data have been deposited in the repository Zenodo. We have added the following line to the end of the legends of every figure:

“The data underlying this Figure can be found in https://doi.org/10.5281/zenodo.7714234”
